# *Streptococcus pneumoniae* induces pyroptosis through the regulation of autophagy in murine microglia

**DOI:** 10.18632/oncotarget.6592

**Published:** 2015-12-13

**Authors:** Ji-Yun Kim, James C. Paton, David E. Briles, Dong-Kwon Rhee, Suhkneung Pyo

**Affiliations:** ^1^ School of Pharmacy, Sungkyunkwan University, Suwon, Kyunggi-do, Republic of Korea; ^2^ School of Molecular and Biomedical Science, University of Adelaide, Adelaide, SA, Australia; ^3^ Department of Microbiology, University of Alabama at Birmingham, Birmingham, AL, USA; ^4^ Department of Genetics, University of Alabama at Birmingham, Birmingham, AL, USA; ^5^ Department of Pediatrics, University of Alabama at Birmingham, Birmingham, AL, USA

**Keywords:** S. pneumoniae, microglia, pyroptosis, autophagy, pneumolysin

## Abstract

*Streptococcus pneumoniae* is responsible for significant mortality and morbidity worldwide and causes invasive pneumococcal diseases including pneumococcal meningitis. Pyroptosis is caspase-1-dependent inflammatory cell death and is known to be induced by various microbial infections. In the present study, we investigated the molecular mechanisms that regulate pyroptosis induced by *S. pneumoniae* in microglia. Our results revealed that *S. pneumoniae* induced pyroptosis through caspase-1 activation and IL-1β production. We also found that the activation of caspase-1 and the maturation of IL-1β and IL-18 in the *S. pneumoniae*-triggered pyroptotic cell death process were mediated by NLRP3 inflammasome. In addition, pneumococcal infection increased the expression of autophagy-related genes and induced autophagosome formation. We also showed that the inhibition of autophagy promoted pneumococcus-induced pyroptosis. Furthermore, ROS was generated by pneumococcal infection and inhibited caspase-1 activation within 4 h of infection. However, in the late phase of infection, IL-1β secretion and caspase-1-dependent cell death were induced by ROS. These results suggest that autophagy induction transiently delay pyroptosis induced by *S. pneumoniae* in microglia. Our study also revealed that the activation of caspase-1 and the production of IL-1β were induced by pneumolysin and that pneumolysin triggered pyroptosis in microglial cells. Similar to the *in vitro* results, *S. pneumoniae* induced caspase-1 activation and caspase-1-dependent cytokine maturation in the mouse meningitis model. Thus, the present data demonstrate that *S. pneumoniae* induces pyroptosis in murine microglia and that NLRP3 inflammasome is critical for caspase-1 activation during the process. Furthermore, the induction of autophagy could transiently protect microglia from pyroptosis.

## INTRODUCTION

*S. pneumoniae* is a Gram-positive, extracellular bacterium that is responsible for high mortality and morbidity worldwide. The most common manifestations of pneumococcal disease include otitis media, pneumonia and meningitis. Meningitis has the worst prognosis of any pneumococcal disease, with a mortality rate of 15∼30 % [1]. The poor prognosis of meningitis is related to the weakness of the host defense mechanisms within the brain, which are notably ineffective in eliminating major meningitis pathogenesis [2]. It has become evident that the host inflammatory reaction to the pathogen, rather than the pathogen itself, is largely responsible for the damage that results from bacterial meningitis [3]. Although the exact mechanisms of immune activation in pneumococcal meningitis remain unclear, recent studies have suggested that the presence of *S. pneumoniae* is recognized by resident immunocompetent cells by means of surface and intracellular pattern recognition receptors such as TLRs and NLRs [4].

Microglia are the resident macrophages of the central nervous system (CNS) and mediate inflammatory responses in the CNS [5]. During pneumococcal infection, cytokines and chemokines are produced by cells lining the brain side of the Blood-Brain Barrier [6], most probably by microglia and astrocytes. The cytokines and chemokines derived from microglia induce the recruitment of leukocytes into the CNS to fight against invading pathogens but coincidentally, contribute to neuronal damage in *S. pneumoniae*-triggered meningitis [7]. Furthermore, it has been reported that *S. pneumoniae* can also directly induce programmed cell death in primary rat hippocampal and cortical neurons, as well as in human microglial and neuronal cell lines during pneumococcal meningitis [8].

Pyroptosis is an inflammatory form of programmed cell death in various types of microbial infection [9] and is distinct from apoptosis since the pathway is inherently dependent on caspase-1 [10]. During the pyroptotic cell death process, caspase-1 is activated by large multiprotein signaling platforms, called inflammasomes [11]. Recently, it has been suggested that pyroptosis is induced by various microorganisms through the activation of different inflammasomes [12]. *Ligionella pneumophila* infection or the secreted flagellin from this pathogen was shown to activate NLRC4-mediated pyroptosis [13, 14], whereas there have been several reports indicating *Listeria monocytogenes* activates multiple inflammasomes, including NLRC4, NLRP3 and AIM2, and trigger pyroptosis [15–17]. In addition, it has been shown that ASC inflammasomes, including AIM2 and NLRP3 activate caspase-1 in pneumococcal infection [18] and especially, pneumolysin, a major virulence factor of *S. pneumoniae*, activate NLRP3/ASC inflammasome in a murine model of *S. pneumoniae* corneal infection [19]. Moreover, recent studies demonstrate that inflammasomes are a key component of the innate immune response in the CNS pathology [20], and only a few inflammasomes have been described and characterized in the CNS, including the NLRP3 inflammasome in microglia [21–23].

Autophagy is a highly conserved homeostatic process for the sequestration and degradation of cytosolic macromolecules and excess or damaged organelles [24]. The capacity to degrade large quantities of cytoplasm also provides cells with a powerful mechanism to degrade intracellular pathogens [25]. A number of bacterial pathogens have been suggested to induce pyroptosis with or without autophagic processes [26, 27]. However, the involvement of autophagy in pneumococcus-induced pyroptotic cell death process has not yet been investigated.

In the present study, we investigated the activation of inflammasome and the subsequent induction of pyroptosis in murine microglial cells during pneumococcal infection. Our findings suggest that pyroptosis is induced by *S. pneumoniae* and that NLRP3 inflammasome regulates the activation of caspase-1 as well as the production of caspase-1-dependent cytokines in pneumococcus-induced pyroptosis. Additionally, we demonstrated that autophagy is triggered by pneumococcal infection and protects microglia from pyroptotic cell death.

## RESULTS

### *S. pneumoniae* induces caspase-1 activation and pyroptosis

**Figure 1:**
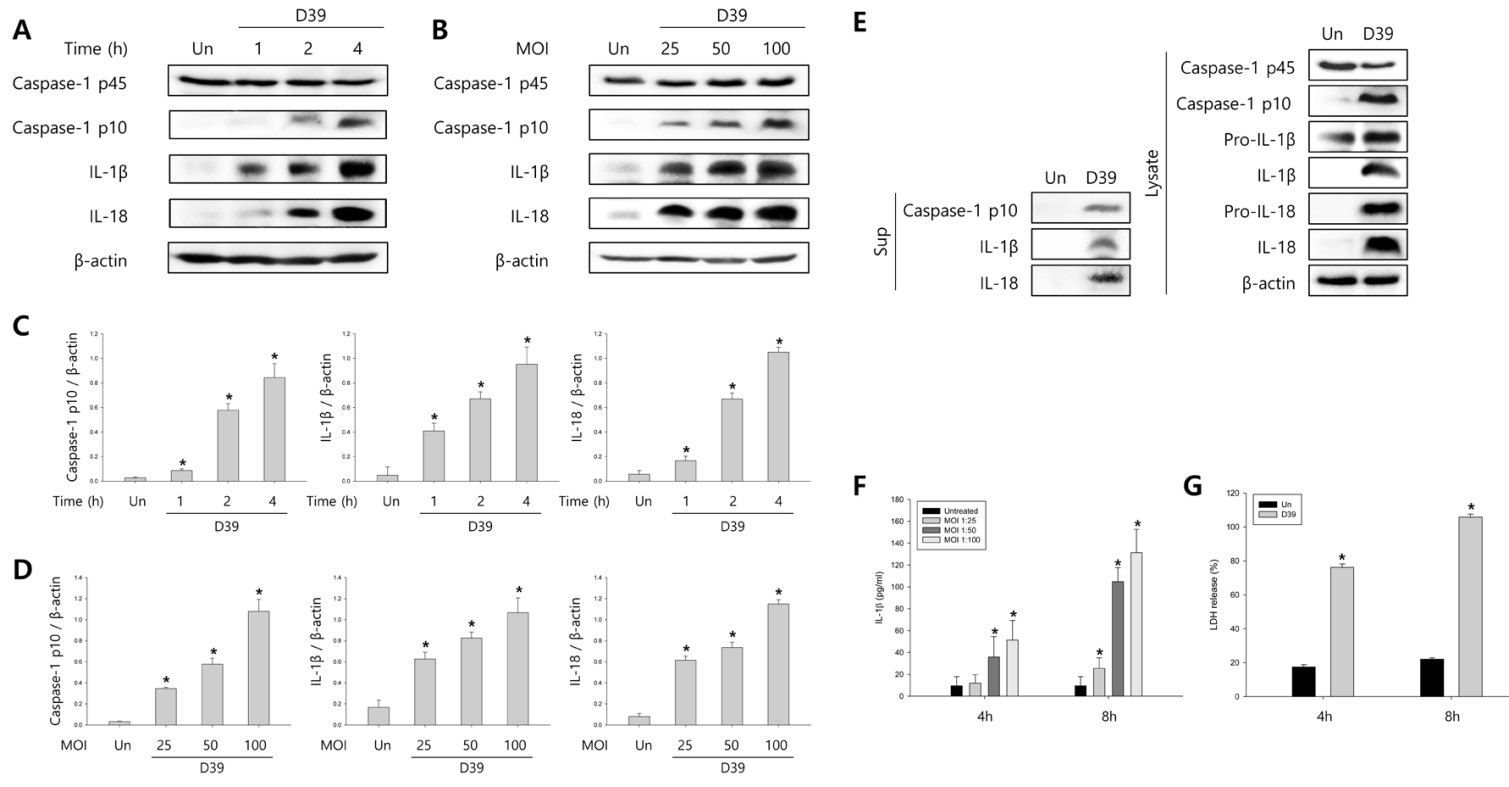

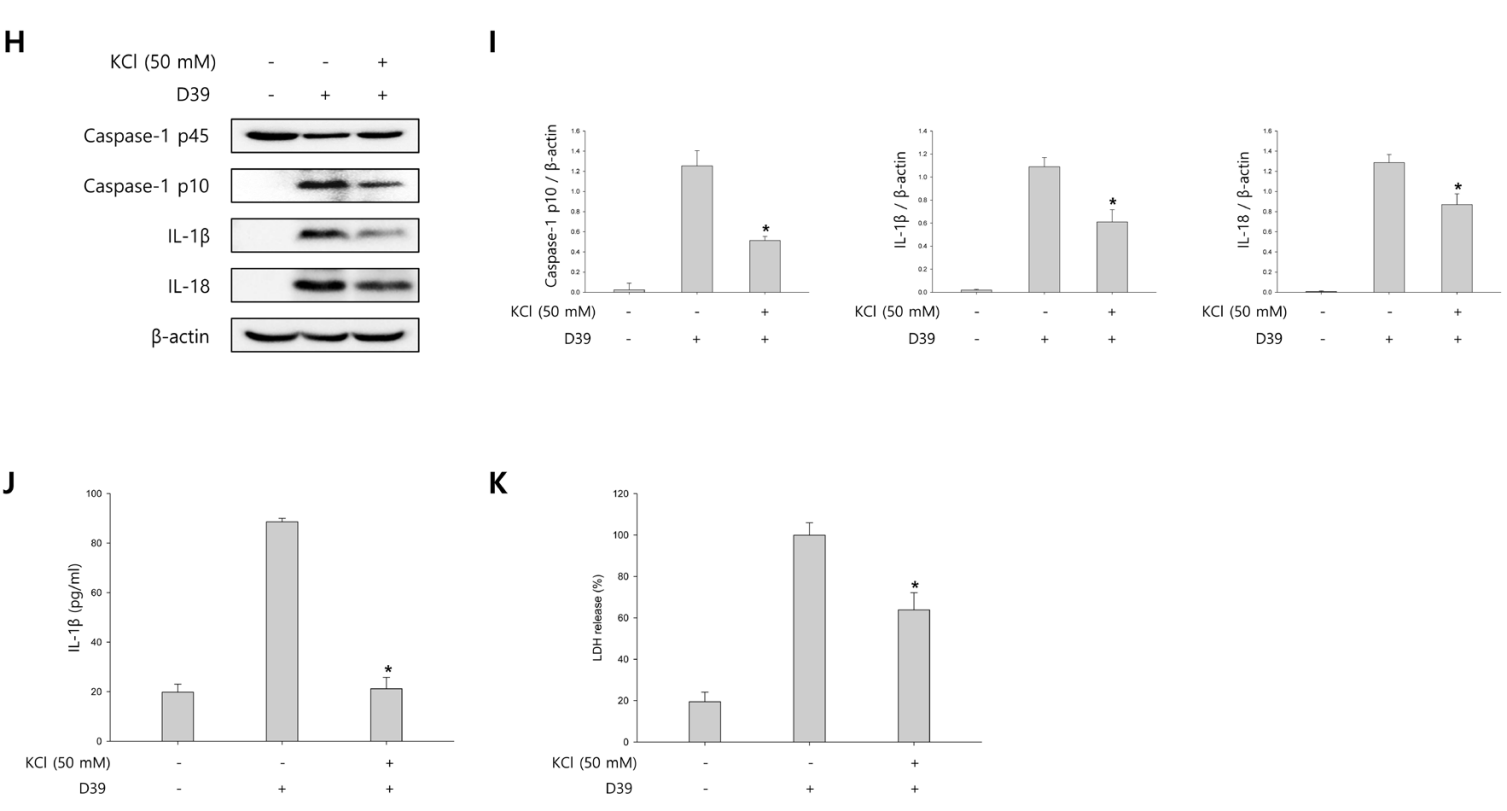
*S. pneumoniae* induces caspase-1 activation and pyroptosis **A.**-**D.** BV-2 microglial cells were left uninfected or were infected with *S. pneumoniae* D39 at an MOI of 100 for 1, 2 and 4 h **A.** and **C.** and at various MOI of 25, 50 and 100 for 4 h **B.** and **D.**, respectively. Cell lysates were subjected to Western blot analysis to detect caspase-1, IL-1β and IL-18. The levels of each protein expression are in arbitrary units, and data are normalized to the respective amount of β-actin protein. These results are representative of three independent experiments with similar results. *p < 0.05, significantly different from uninfected control. **E.** BV-2 cells were left uninfected or were infected with D39 at an MOI of 100 for 4 h. Culture supernatants were concentrated by trichloroacetate precipitation. Concentrated supernatants and cell lysates were subjected to Western blot analysis using antibodies specific for caspase-1, IL-1β, IL-18 and β-actin. β-actin was used as a loading control. These results are representative of three independent experiments. **F.** Microglial cells were infected with D39 at various MOI of 25, 50 and 100 for 4 h and 8 h. The levels of IL-1β in the culture supernatants were determined by ELISA. Data are expressed as the mean ± S.E.M. of 3 experiments. *p < 0.05, significantly different from uninfected control. **G.** BV-2 microglial cells were infected with D39 at an MOI of 100 for 4 h and 8 h. LDH release into the culture media is shown as a percentage of LDH release. Data are expressed as the mean ± S.E.M. of 3 experiments. *Significantly different from uninfected cells (*p* < 0.05). **H.**-**K.** Potassium efflux is required for *S. pneumoniae*-induced pyroptosis. **H.** and **I.** BV-2 microglial cells were infected with *S. pneumoniae* D39 at an MOI of 100 in the presence or absence of 50 mM of KCl for 4 h. Cell lysates were subjected to Western blot analysis to detect caspase-1 and caspase-1-dependent cytokines. The levels of each protein expression are in arbitrary units, and data are normalized to the respective amount of β-actin protein. These results are representative of three independent experiments with similar results. *p < 0.05, significantly different from *S. pneumoniae*-infected control. **J.** and **K.** BV-2 cells were infected with D39 at an MOI of 100 in the presence or absence of 50 mM of KCl for 8 h. The levels of IL-1β in the culture supernatants were determined by ELISA **J.**. LDH release into the culture media is shown as a percentage of LDH release **K.**. Data are expressed as the mean ± S.E.M. of 3 experiments. *p < 0.05, significantly different from *S. pneumoniae*-infected control.

To investigate whether *S. pneumoniae* activates caspase-1 and matures caspase-1-dependent cytokines, BV-2 microglial cells were infected with *S. pneumoniae* D39 at various MOI of 25, 50 and 100 for various time points. As shown in Figure 1A–1D, caspase-1 was activated and cleaved time- and concentration-dependently by D39 infection. The expression of caspase-1-dependent cytokines, IL-1β and IL-18, was also increased in a time- and concentration-dependent manner. Furthermore, significant levels of the p10 fragment of mature caspase-1, mature IL-1β and IL-18 were released into the culture supernatant (Figure 1E). In order to examine *S. pneumoniae*-induced cytokine secretion, microglial cells were infected with D39 for 4 or 8 h and the levels of IL-1β, IL-6 and TNF-α in the culture media were determined by ELISA. The production of IL-1β was significantly increased by D39 in a concentration-dependent manner (Figure 1F). A significant increase of IL-6 and TNF-α were also observed at 4 or 8 h following infection with D39 or TIGR4 (Supplementary Figure S2). We further investigated whether *S. pneumoniae* induces inflammatory cell death. As shown in Figure 1G, a significant amount of LDH was released and increased time-dependently by pneumococcal infection. Since depletion of intracellular potassium is shown to induce pyroptosis and IL-1β secretion (Warny and Kelly, 1999 and Walev et al., 1995), we investigated the effect of potassium efflux on *S. pneumoniae*-induced pyroptosis by increasing the extracellular potassium concentration. As shown in Figure 1H–1I, *S. pneumoniae* D39-induced caspase-1 activation was decreased by high extracellular potassium. The inhibition of potassium efflux also reduced the expression of caspase-1-dependent cytokines, IL-1β and IL-18. Additionally, high extracellular potassium blocked IL-1β secretion and LDH release from D39-infected BV-2 cells (Figure 1J–1K). Similar results were obtained from BV-2 cells incubated with *S. pneumoniae* TIGR4 and C6 glioma cells infected with *S. pneumoniae* D39 (Supplementary Figure 1 and Supplementary Figure 3). Collectively, these findings suggest that *S. pneumoniae* induces the activation of caspase-1, the production of caspase-1-dependent cytokines and pyroptosis in BV-2 microglial cells.

### NLRP3 inflammasome is involved in *S. pneumoniae*-induced caspase-1 activation

**Figure 2:**
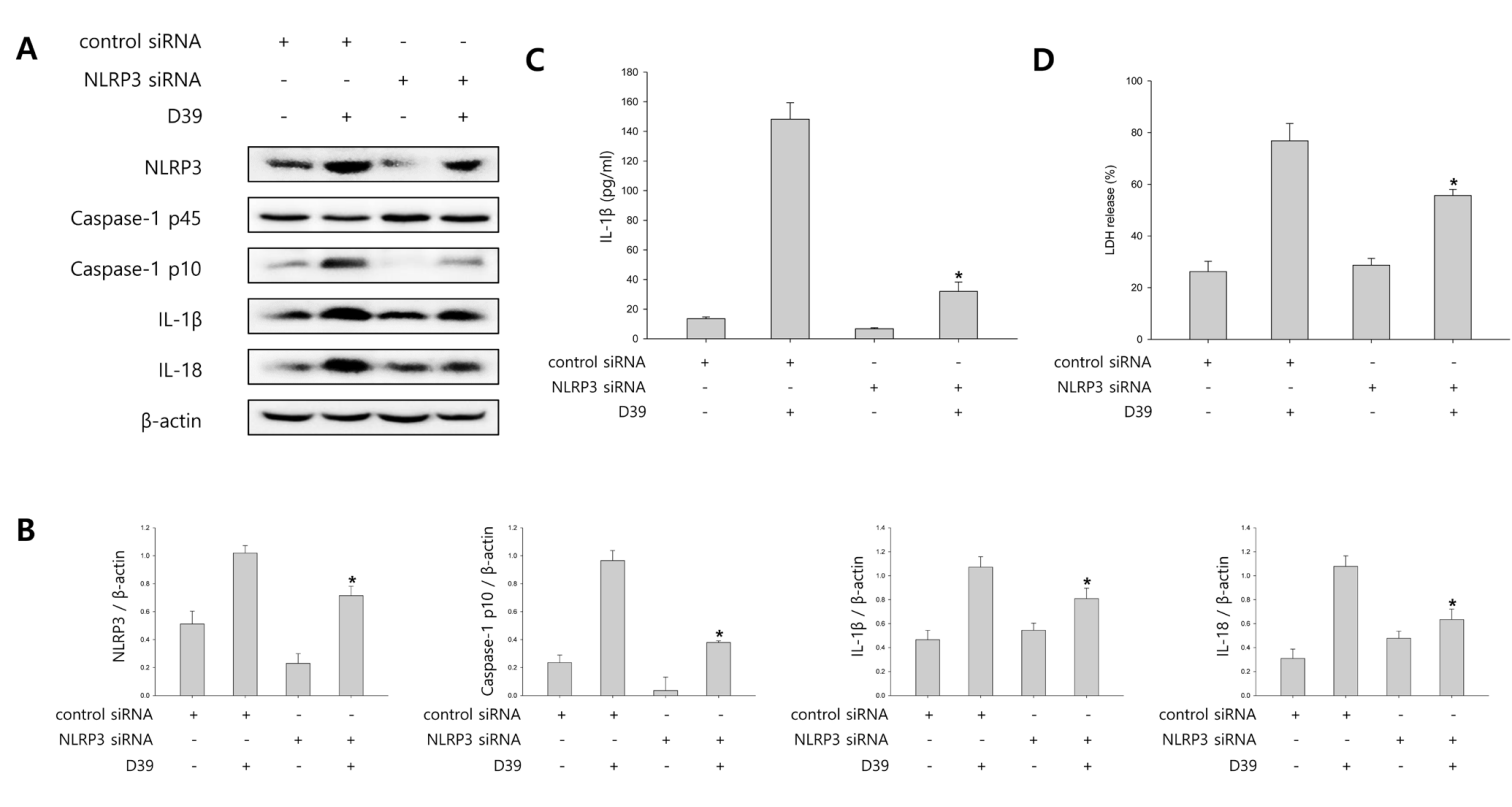
NLRP3 inflammasome is involved in *S. pneumoniae*-induced caspase-1 activation **A.** and **B.** BV-2 cells were transfected with 100 nM of control siRNA or NLRP3 specific siRNA for 24 h and infected with *S. pneumoniae* D39 at an MOI of 100 for 2 h (NLRP3) and 4 h (caspase-1, IL-1β and IL-18). β-actin was used as a loading control. The levels of each protein expression are in arbitrary units, and data are normalized to the respective amount of β-actin protein. These results are representative of three independent experiments with similar results. *p < 0.05, significantly different from control siRNA-transfected and *S. pneumonie* D39-infected control. **C.** and **D.** BV-2 cells were transfected with 100 nM of control siRNA or NLRP3 specific siRNA for 24 h and infected with D39 at an MOI of 100 for 8 h. **C.** The levels of IL-1β in the culture supernatants were determined by ELISA. **D.** The levels of LDH in the culture media are shown as a percentage of LDH release. These results are representative of three independent experiments with similar results. *p < 0.05, significantly different from control siRNA-transfected and *S. pneumoniae* D39-infected control.

To determine the contribution of NLRP3 inflammasome to caspase-1 activation and caspase-1-dependent cytokine maturation, we carried out siRNA mediated knockdown of NLRP3. As shown in Figure 2A–2B, NLRP3 siRNA treatment resulted in a significant decrease in cleaved caspase-1 expression as well as in IL-1β and IL-18 expression in D39-infected microglial cells. In addition, we examined whether *S. pneumoniae*-induced production of IL-1β is regulated by NLRP3 inflammasome. Figure 2C showed that treatment with siNLRP3 significantly decreased the level of IL-1β in the supernatant of D39-infected cells. Subsequently, to investigate whether NLRP3 inflammasome is responsible for *S. pneumoniae*-induced inflammatory cell death, siRNA (control siRNA or NLRP3 siRNA)-transfected microglial cells were infected with D39 and the levels of LDH in the culture media was then determined. As shown in Figure 2D, NLRP3 siRNA treatment significantly reduced the LDH release induced by *S. pneumoniae* D39. These results suggest that NLRP3 inflammasome regulates the activation of caspase-1 in response to pneumococcal infection.

### Autophagy inhibits *S. pneumoniae*-induced caspase-1 activation and pyroptosis

**Figure 3:**
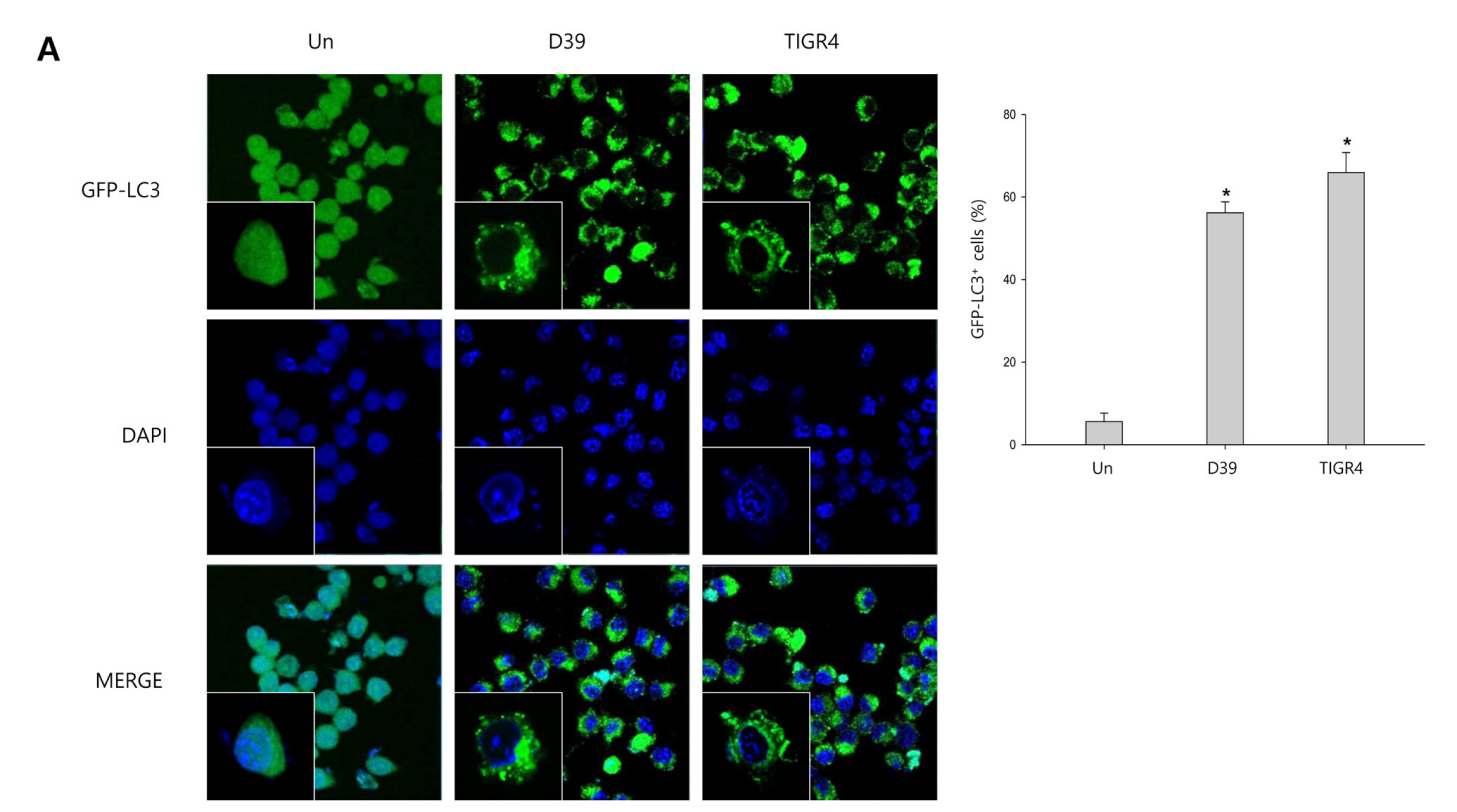

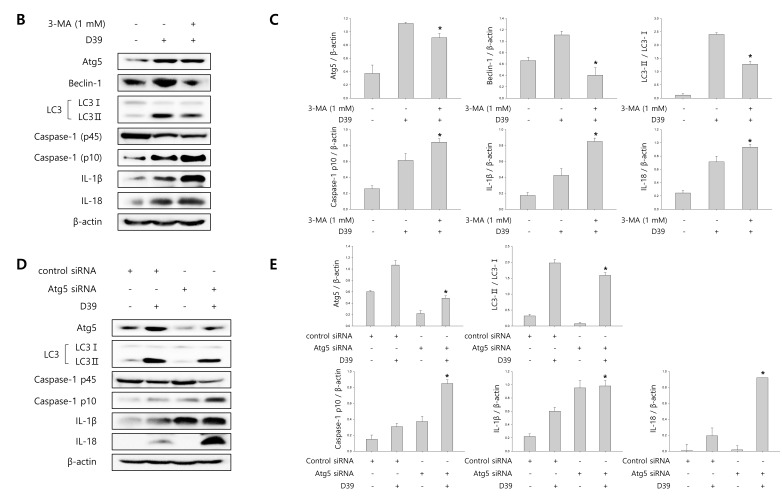

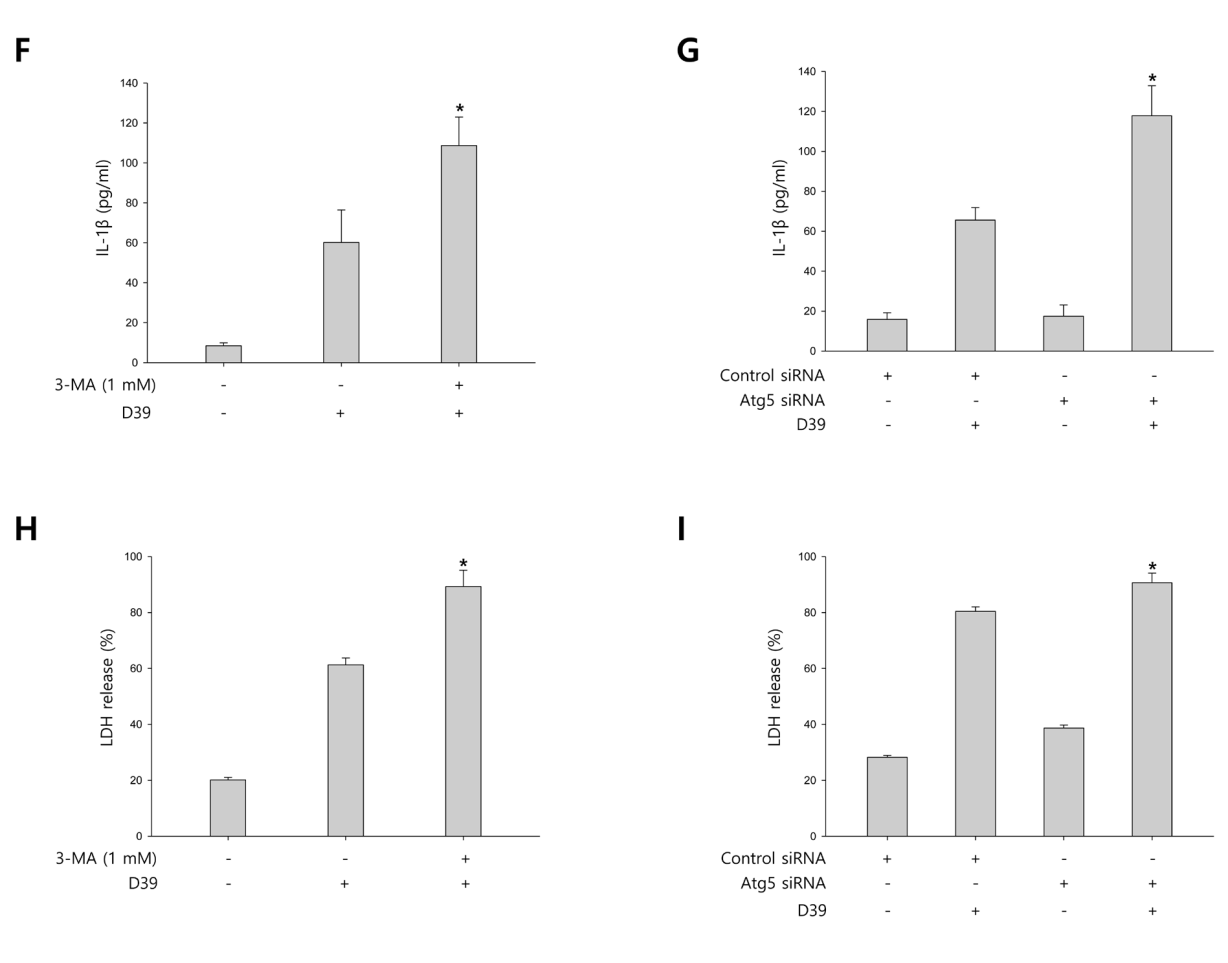
Autophagy inhibits *S. pneumoniae*-induced caspase-1 activation and pyroptosis **A.** BV-2 cells were transfected with GFP-LC3 for 24 h and infected with *S. pneumoniae* D39 or TIGR4 at an MOI of 100 for 4 h. GFP-LC3 expressing cells were visualized with a fluorescence microscope and the results are presented as the quantification of GFP-LC3^+^ dots. These results are representative of three independent experiments with similar results. *p < 0.05, significantly different from the uninfected control. **B.** and **C.** BV-2 microglial cells were left uninfected or were infected with D39 at an MOI of 100 for 2 h (Atg5 and Beclin-1) and 4 h in the presence or absence of 3-MA (1 mM). Cell lysates were subjected to Western blot analysis. The levels of each protein expression are in arbitrary units, and data are normalized to the respective amount of β-actin protein. The LC3 I and LC3 II bands were quantified by densitometry. These results are representative of three independent experiments with similar results. *p < 0.05, significantly different from *S. pneumoniae* D39-infected control. **D.** and **E.** BV-2 cells were transfected with 50 nM of control siRNA or Atg5 siRNA for 24 h and infected with D39 at an MOI of 100 for 2 h (Atg5 and Beclin-1) and 4 h. Cell lysates were subjected to Western blot analysis. The levels of each protein expression are in arbitrary units, and data are normalized to the respective amount of β-actin protein. The LC3 I and LC3 II bands were quantified by densitometry. These results are representative of three independent experiments with similar results. *p < 0.05, significantly different from control siRNA-transfected and *S. pneumoniae* D39-infected control. **F.** and **H.** BV-2 microglial cells were left uninfected or were infected with D39 at an MOI of 100 for 8 h in the presence or absence of 3-MA (1 mM). **G.** and **I.** BV-2 cells were transfected with 50 nM of control siRNA or Atg5 siRNA for 24 h and infected with D39 at an MOI of 100 for 8 h. The levels of IL-1β in the culture supernatants were determined by ELISA **F.** and **G.**. The levels of LDH in the culture media are shown as a percentage of LDH release **H.** and **I.**. These results are representative of three independent experiments with similar results. *p < 0.05, significantly different from *S. pneumoniae* D39-infected control.

To investigate whether *S. pneumoniae* induces autophagy, we determined the expression of several autophagy-related proteins by Western blot analysis. As shown in Supplementary Figure 4, the expression of Atg5 and beclin-1 was rapidly increased by *S. pneumoniae* D39. The level of LC3 II was time-dependently increased in D39-infected cells. Interestingly, the expression of NLRP3 was also rapidly increased and remained elevated until 4 h of infection. In addition, the expression of IL-1β, IL-18 and caspase-1 p10 was gradually increased and remained elevated until 7 h of infection. These results demonstrate that autophagy is induced in the early phase (4 h) of pneumococcal infection, before pyroptosis occurs. Furthermore, as shown in Figure 3A, LC3 autophagosomes were observed in the cells infected with *S. pneumoniae* D39 or TIGR4. Next, we examined the role of autophagy in the *S. pneumoniae*-induced activation of caspase-1 and the subsequent maturation of caspase-1-dependent cytokines. BV-2 cells were incubated with 3-methyladenine (3-MA), a well-known inhibitor of autophagy, for 2 h and then infected with *S. pneumoniae* D39. As shown in Figure 3B–3C, D39-induced expression of Atg5, beclin-1 and LC3 II was decreased by 3-MA treatment. In addition, the treatment with 3-MA reduced LC3 autophagosome formation (Supplementary Figure 5A). On the contrary, the elevated expression of caspase-1 p10, IL-1β and IL-18 by D39 infection was further increased in response to treatment with 3-MA (Figure 3B–3C). Moreover, the production of IL-1β was significantly elevated by D39-infection, whereas 3-MA treatment further increased IL-1β release from *S. pneumoniae* D39-infected BV-2 cells (Figure 3F). To confirm whether the inhibition of autophagy enhances caspase-1 activation and subsequent cytokine maturation in *S. pneumoniae* D39-infected microglial cells, siRNA mediated knockdown of Atg5 was performed. As shown in Figure 3D–3E, treatment with siAtg5 significantly decreased Atg5 protein expression and endogenous LC3 I to LC3 II conversion. In addition, GFP-LC3 autophagosomes were reduced by siAtg5 treatment (Supplementary Figure 5B). However, the expression of cleaved caspase-1 was significantly elevated by D39 infection, and treatment with siAtg5 further increased the expression levels. Additionally, Atg5 siRNA treatment increased the expression of IL-1β and IL-18 in D39-infected cells compared to the non-specific siRNA-transfected control (Figure 3D–3E). Moreover, consistent with Figure 3F, treatment with Atg5 siRNA significantly increased the level of IL-1β in the cell culture media compared to the culture media for the control siRNA-transfected cells (Figure 3G). Next, we investigated whether autophagy inhibits *S. pneumoniae*-induced pyroptosis. As shown in Figure 3H, *S. pneumoniae* D39 significantly induced LDH release and 3-MA treatment further increased the release of LDH. Figure 3I also shows that the release of LDH was significantly induced by *S. pneumoniae* D39, whereas knockdown of Atg5 released more LDH in response to D39 infection. To further confirm whether autophagy inhibits pyroptosis in *S. pneumoniae* D39-infected microglial cells, BV-2 cells were incubated with actinomycin D (ACT-D), a general transcriptional inhibitor used for inhibiting autophagy induced by ER-stress, or bafilomycin A1 (BAF-A1), a well-known inhibitor of the late phase of autophagy, for 2 h prior to D39 infection. As shown in Supplementary Figure 6A-6D, *S. pneumoniae* D39 significantly increased the expression of cleaved caspase-1 and caspae-1-dependent cytokines in microglial cells and pretreatment with ACT-D or BAF-A1 further increased cleaved caspase-1 and cytokine expression. In addition, the levels of IL-1β and LDH in the cell culture media were further increased by ACT-D and BAF-A1 treatment, respectively (Supplementary Figure 6E-6H). Taken together, these results suggest that autophagy inhibits caspase-1 activation and pyroptosis in *S. pneumoniae* D39-infected BV-2 microglial cells.

### ROS regulates *S. pneumoniae*-induced pyroptosis through the activation of autophagy

**Figure 4:**
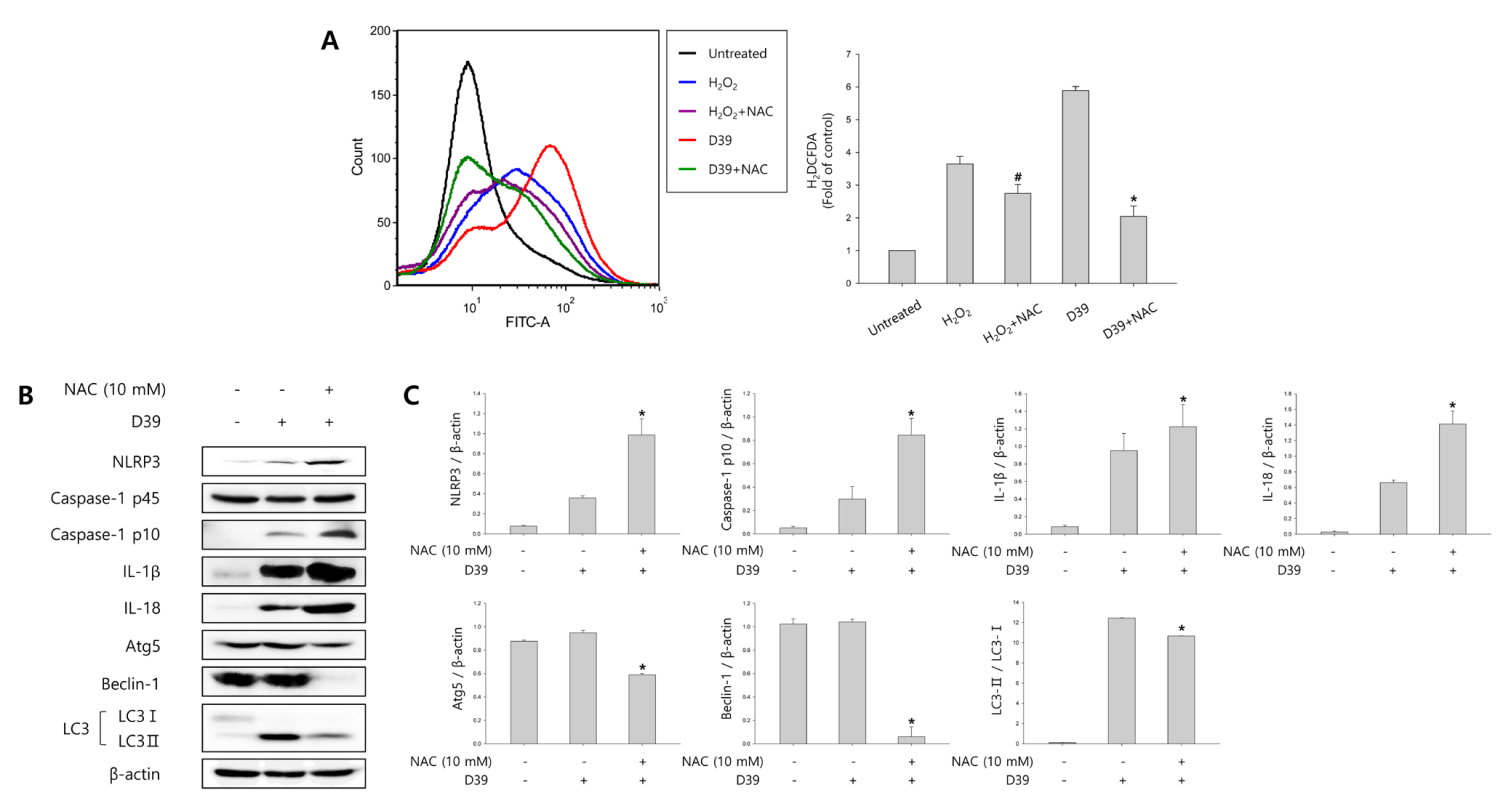

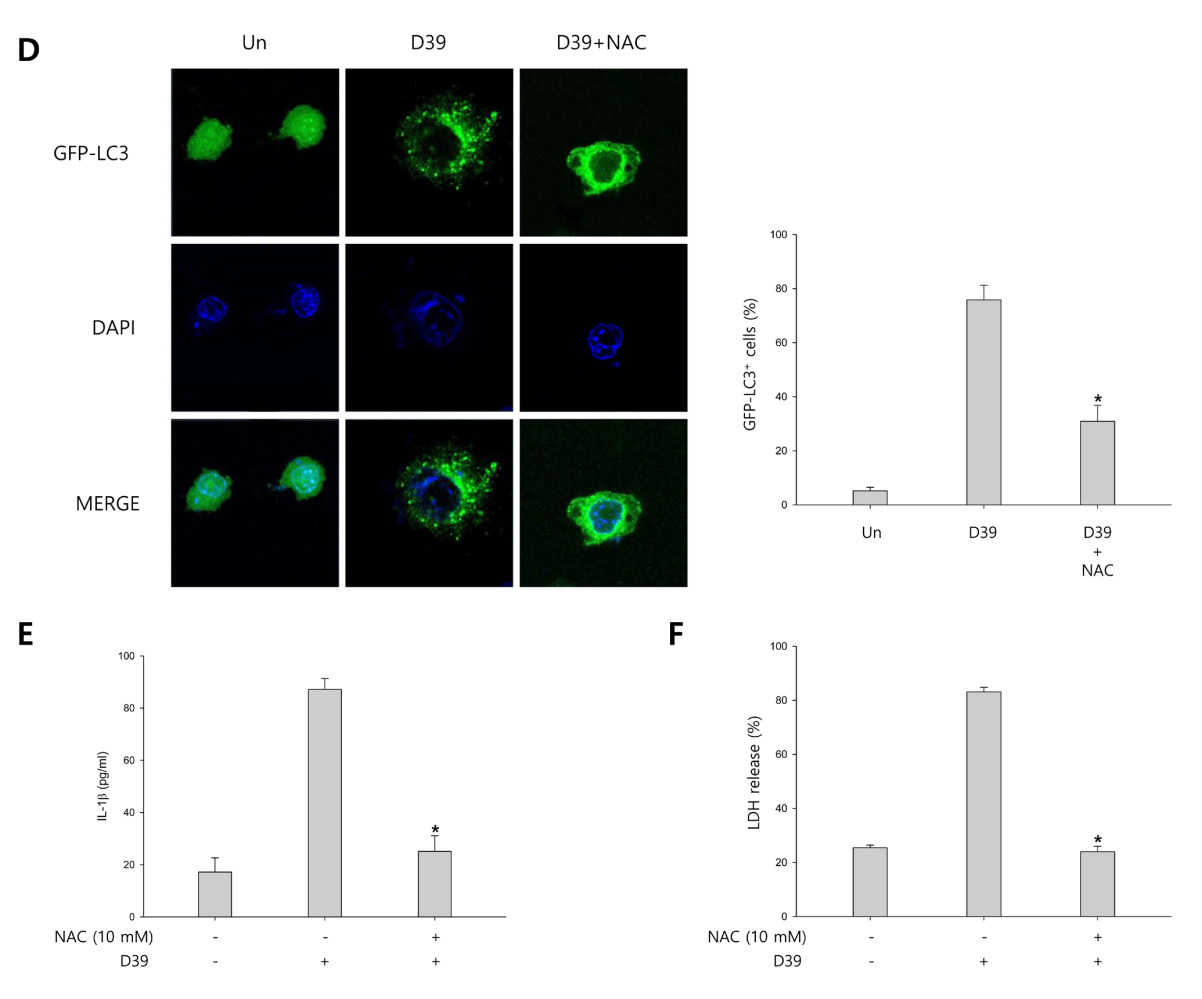
ROS regulates *S. pneumoniae*-induced pyroptosis through the activation of autophagy **A.** BV-2 microglial cells were treated with H_2_O_2_ (10 mM) or infected with *S. pneumoniae* D39 at an MOI of 100 for 2 h in the presence or absence of NAC (10 mM) then stained for 5 min with H2DCFDA (50 μM). The generation of ROS was analyzed by flow cytometry. DCF fluorescence was quantified. These results are representative of three independent experiments. ^#^*p* < 0.05, significantly different from H_2_O_2_-treated control. *p < 0.05, significantly different from *S. pneumoniae* D39-infected control. **B.** and **C.** BV-2 microglial cells were infected with *S. pneumoniae* D39 at an MOI of 100 for 2 h (NLRP3, Atg5 and Beclin-1) and 4 h (Caspase-1, IL-1β, IL-18 and LC3) in the presence or absence of NAC (10 mM). Cell lysates were subjected to Western blot analysis. The levels of each protein expression are in arbitrary units, and data are normalized to the respective amount of β-actin protein. These results are representative of three independent experiments with similar results. *p < 0.05, significantly different from *S. pneumoniae* D39-infected control. **D.** BV-2 cells were transfected with GFP-LC3 for 24 h and infected with *S. pneumoniae* D39 in the presence or absence of NAC (10 mM) at an MOI of 100 for 4 h. GFP-LC3 expressing cells were visualized with a fluorescence microscope and the results are presented as the quantification of GFP-LC3^+^ dots. These results are representative of three independent experiments with similar results. *p < 0.05, significantly different from *S. pneumoniae* D39-infected control. **E.** and **F.** Microglial cells were left uninfected or infected with D39 at an MOI of 100 for 8 h in the presence or absence of NAC (10 mM). The levels of IL-1β in the culture supernatants were determined by ELISA **E.**. The levels of LDH in the culture media are shown as a percentage of LDH release **F.**. These results are representative of three independent experiments with similar results. *p < 0.05, significantly different from *S. pneumoniae* D39-infected control.

We investigated whether *S. pneumoniae* D39 generates ROS in D39-infected microglial cells. N-acetyl cysteine (NAC), a widely used thiol-containing antioxidant, was used to inhibit ROS production. As shown in Figure 4A, intracellular ROS generation was significantly increased by D39 infection and NAC significantly inhibited the level of ROS in response to D39 infection. To examine whether autophagy is stimulated by ROS, the expression of autophagy-related proteins was examined by immunoblotting. As shown in Figure 4B–4C, the elevated expression of Atg5 and beclin-1 by D39 infection was significantly decreased by NAC treatment. The level of LC3 II was also reduced by treatment with NAC. In addition, NAC treatment decreased the formation of GFP-LC3 autophagosomes in *S. pneumoniae* D39-infected BV-2 cells (Figure 4D). Next, to determine the role of ROS in the activation of caspase-1 and the maturation of caspase-1-dependent cytokines, the expression of caspase-1, IL-1β and IL-18 in D39-infected BV-2 microglial cells was also examined. As shown in Figure 4B–4C, caspase-1 was activated and the level of cleaved caspase-1 was increased within 4 h of pneumococcal infection. Additionally, treatment with NAC further increased the expression of cleaved caspase-1. Consistent with caspase-1 activation, the elevated levels of IL-1β and IL-18 in D39-infected microglia were further increased by NAC treatment. We also investigated the role of *S. pneumoniae* D39-induced ROS on the production of IL-1β at the late phase (8 h) of infection. Interestingly, the production of IL-1β was significantly increased by *S. pneumoniae* D39 infection, whereas treatment with NAC significantly reduced IL-1β production (Figure 4E). To assess the role of ROS in pyroptotic cell death, BV-2 cells were infected with *S. pneumoniae* D39 in the presence or absence of NAC and the LDH concentration in the culture media was measured. As shown in Figure 4F, the increased level of LDH in the culture supernatant of D39-infected cells was significantly reduced by NAC treatment. Taken together, our results demonstrate that *S. pneumoniae* D39-induced ROS generation activates autophagy at 4 h post-infection and also induces the production of IL-1β and the release of LDH at the late phase (8 h) of pneumococcal infection, leading to cell death.

### Pneumolysin induces caspase-1 activation and pyroptosis

**Figure 5:**
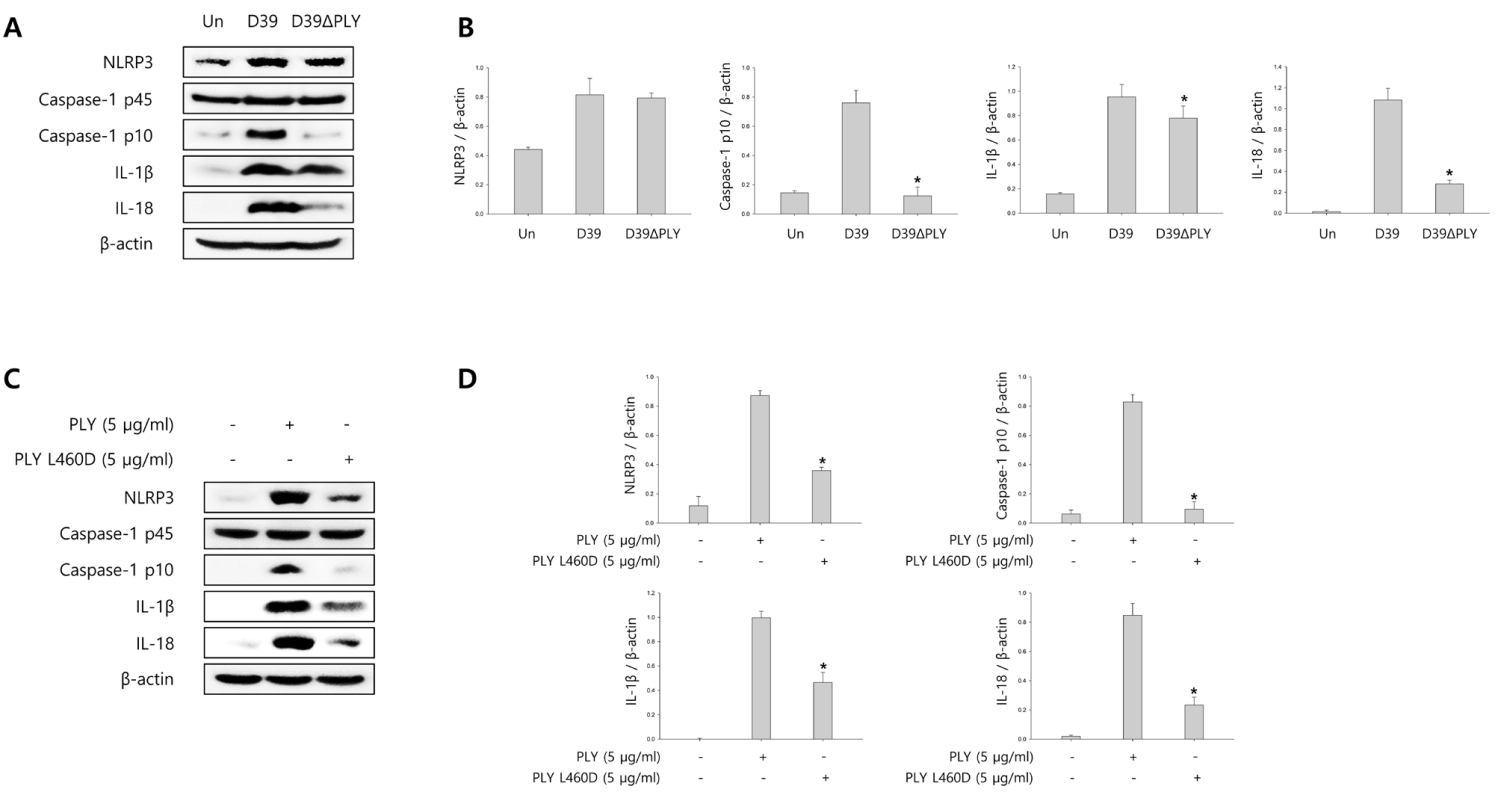

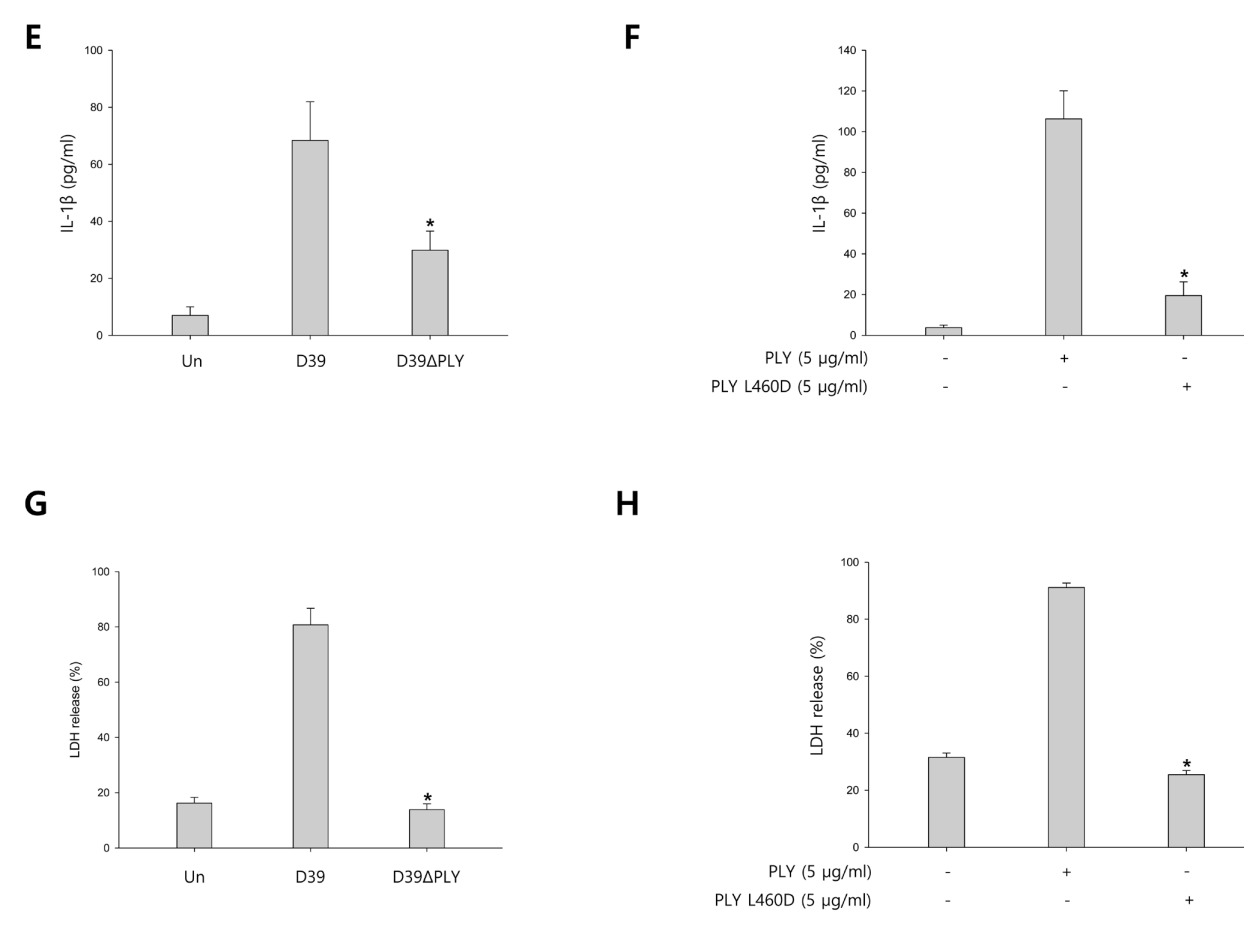
Pneumolysin induces caspase-1 activation and pyroptosis **A.** and **B.** BV-2 cells were infected with *S. pneumoniae* D39 or D39ΔPLY for 2 h (NLRP3) and 4 h. The levels of each protein expression are in arbitrary units, and data are normalized to the respective amount of β-actin protein. These results are representative of three independent experiments with similar results. *p < 0.05, significantly different from *S. pneumoniae* D39-infected control. **C.** and **D.** Microglial cells were treated with pneumolysin (PLY) or pneumolysin L460D (PLY L460D) for 6 h (NLRP3) and 12 h. The levels of each protein expression are in arbitrary units, and data are normalized to the respective amount of β-actin protein. These results are representative of three independent experiments with similar results. *p < 0.05, significantly different from PLY-treated control. **E.** and **G.** BV-2 cells were infected with *S. pneumoniae* D39 or D39ΔPLY at an MOI of 100 for 8 h. **F.** and **H.** BV-2 cells were treated with PLY or PLY L460D for 24 h. The levels of IL-1β in the culture supernatants were determined by ELISA **E.** and **F.**. The levels of LDH in the culture media are shown as a percentage of LDH release **G.** and **H.**. These results are representative of three independent experiments with similar results. *p < 0.05, significantly different from *S. pneumoniae* D39-infected control or PLY-treated control.

To investigate whether pneumolysin (PLY) activates caspase-1 and matures caspase-1-dependent cytokines, BV-2 cells were infected with *S. pneumoniae* D39 or its PLY-deficient mutant, D39ΔPLY for 2 h and 4 h. As shown in Figure 5A–5B, the expression of cleaved caspase-1 was significantly increased by wild-type D39 infection, whereas D39ΔPLY induced less increase of caspase-1 p10 expression. Furthermore, the levels of NLRP3 and caspase-1-dependent cytokines were less increased in PLY mutant infected cells than in wild-type infected cells. BV-2 microglial cells were also treated with 0.5, 1 and 5 μg/ml of PLY, respectively. As shown in Supplementary Figure 7A–7B, the mature fragment of caspase-1, NLRP3 and caspase-1-dependent cytokines were increased in a concentration-dependent manner. In addition, we determined the effect of PLY on caspase-1 activation by comparing wild-type PLY to the detoxified derivative of PLY, pneumolysin L460D (PLY L460D). As shown in Figure 5C–5D, the expression of NLRP3, caspase-1 p10, IL-1β and IL- 18 was less increased in PLY L460D-treated cells compared to those treated with wild-type PLY. Moreover, the levels of IL-1β in the culture media of D39ΔPLY-infected cells were significantly lower than those of wild-type-infected cells (Figure 5E). Supplementary Figure 7C shows that IL-1β production was significantly increased by PLY in a concentration-dependent manner. Furthermore, PLY L460D significantly reduced the production of IL-1β compared to that induced by wild-type PLY (Figure 5F). To investigate whether pyroptotic cell death is induced by PLY, the levels of LDH in D39 or D39ΔPLY-infected microglial cells were determined. As shown in Figure 5G, wild-type D39-infected cells released more LDH into the culture media than D39ΔPLY-infected cells. A significant amount of LDH was also released by PLY treatment and increased in a concentration- and time-dependent manner (Supplementary Figure 7D). In addition, Figure 5H indicates that PLY L460D failed to induce the release of LDH. Collectively, these results suggest that pneumolysin plays an important role in caspase-1 activation, IL-1β production and subsequent pyroptosis in *S. pneumoniae* D39-infected BV-2 microglial cells.

### *S. pneumoniae* induces caspase-1 activation in murine pneumococcal meningitis

**Figure 6:**
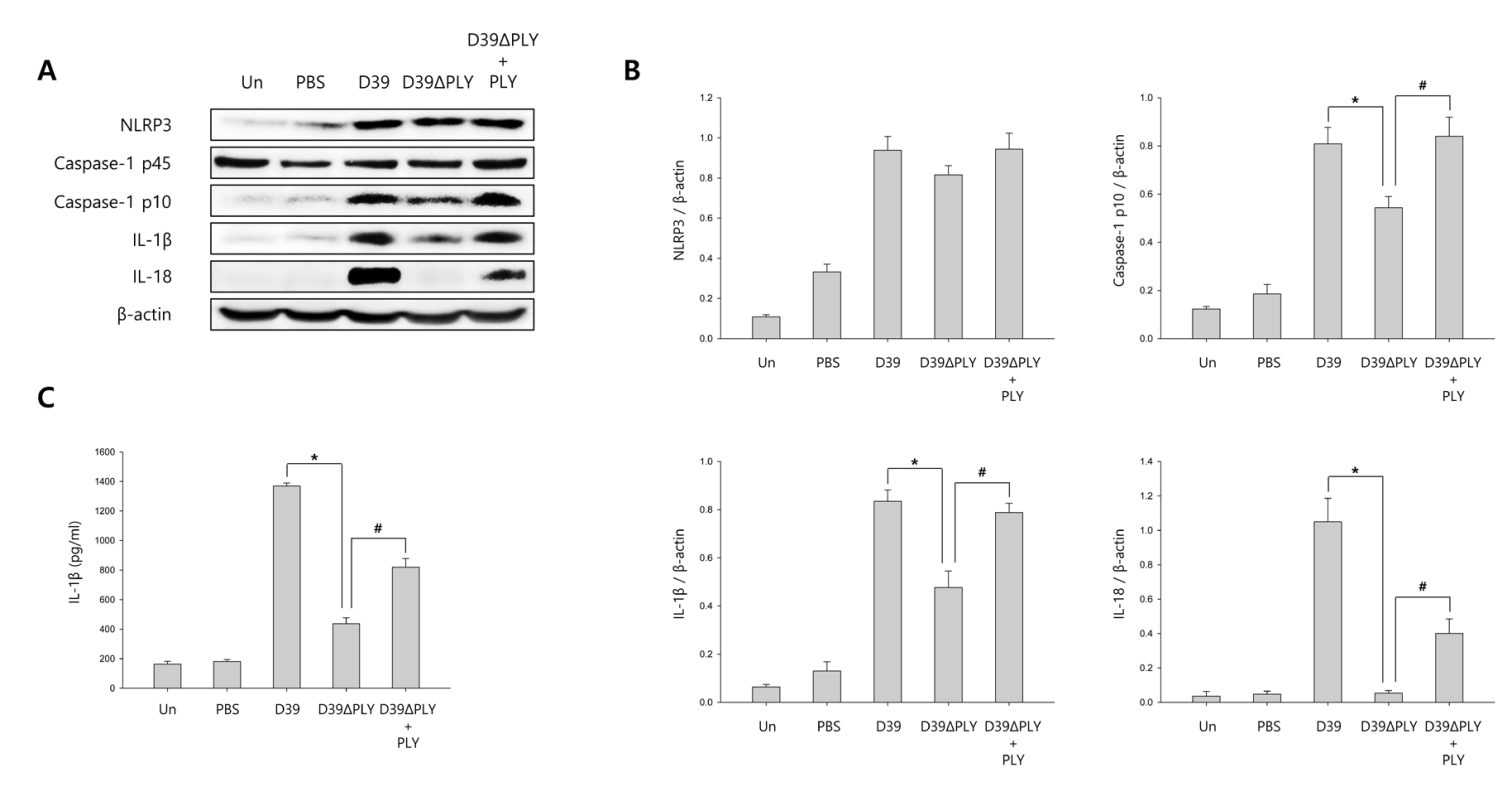

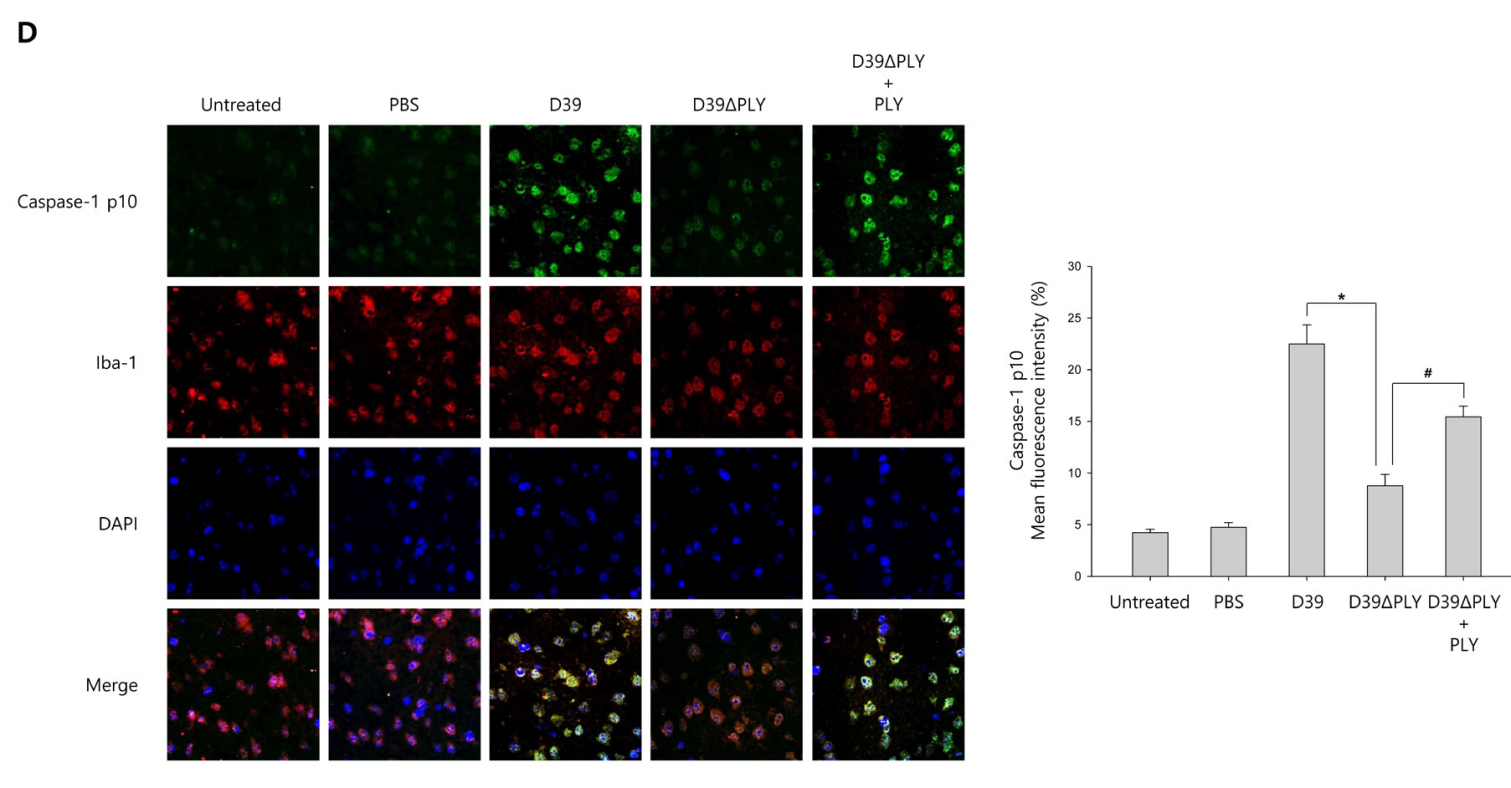
*S. pneumoniae* induces caspase-1 activation in murine pneumococcal meningitis **A.** and **B.** C57BL/6 mice were challenged with control PBS, *S. pneumoniae* D39, the pneumolysin-deficient mutant of D39, D39ΔPLY or D39ΔPLY supplemented with recombinant PLY (200 ng/g) *via* intracerebral ventricular injection. The protein expression of NLRP3, Caspase-1, IL-1β and IL-18 in brain homogenates was determined by Western blot analysis. The levels of each protein expression are in arbitrary units, and data are normalized to the respective amount of β-actin protein. These results are representative of three independent experiments with similar results. *p < 0.05, significantly different from *S. pneumoniae* D39-infected control. ^#^*p* < 0.05, significantly different from D39ΔPLY-infected control. **C.** The levels of IL-1β in brain homogenates were determined by ELISA. These results are representative of three independent experiments with similar results. *p < 0.05, significantly different from *S. pneumoniae* D39-infected control. ^#^*p* < 0.05, significantly different from D39ΔPLY-infected control. **D.** Immunohistochemical analysis of caspase-1 in *S. pneumoniae*-infected mouse brains. Sections were stained for caspase-1 p10 (FITC) and the glial cell marker, Iba-1 (Texas Red) (x400).

To investigate whether *S. pneumoniae* activates caspase-1 and matures caspase-1-dependent cytokines in *S. pneumoniae*-infected mice, pneumococcal meningitis was induced by intracerebral injection of a bacterial suspension containing 10^6^ CFU/ml of *S. pneumoniae* D39, D39ΔPLY or TIGR4 supplemented with or without recombinant PLY protein into the lateral ventricle. As shown in Figure 6A–6B and Supplementary Figure 8A-8B, caspase-1 was activated and cleaved in brain homogenates at 24 h after infection in *S. pneumoniae* D39- or TIGR4-challenged mice, unlike in PBS-treated control mice. The expression of caspase-1-dependent cytokines, IL-1β and IL-18, was also elevated in the brain of D39- or TIGR4-infected mice. However, D39ΔPLY infection caused a reduced level of capase-1 p10 and the expression of IL-1β and IL-18 was also less elevated in the brains of D39ΔPLY-challeged mice than in D39-challenged mice. Interestingly, the expression of cleaved caspase-1 and caspase-1-dependent cytokines was rescued by the addition of recombinant PLY protein. We also determined the levels of IL-1β in brain homogenates by ELISA. As shown in Figure 6C and Supplementary Figure 8C, a significantly increased expression of IL-1β was observed in the brain homogenates of *S. pneumoniae* D39- or TIGR4-infected mice, whereas the levels of IL-1β were significantly reduced in D39ΔPLY-infected mice compared to those of wild-type D39-infected mice. Besides, the supplementation with PLY rescued the infectivity of D39ΔPLY and resulted in increased IL-1β levels. In addition, the expression of caspase-1 in the brains of *S. pneumoniae*-infected mice was examined by immunohistochemistry analysis. As shown in Supplementary Figure 8D, the expression of caspase-1 was highly increased in D39- or TIGF4-challenged mice. Especially, D39 infection resulted in greater increases in the level of cleaved caspase-1, whereas it was decreased in D39ΔPLY-infected mice (Figure 6D). The decreased expression of caspase-1 p10 was recovered by the addition of PLY in D39ΔPLY-infected mice (Figure 6D). Collectively, our results demonstrate that *S. pneumoniae*, especially pneumolysin, induces the activation of caspase-1 and the release of IL-1β in murine pneumococcal meningitis.

## DISCUSSION

*Streptococcus pneumoniae* is the leading cause of bacterial meningitis. During microbial infection, caspase-1 is activated by inflammasomes and promotes pyroptosis, which is crucial for controlling microbial infections [10]. In the present study, we identified the molecular mechanisms that regulate cell death induced by *S. pneumoniae* in microglia. We found that pyroptosis is triggered by *S. pneumoniae* in BV-2 microglial cells and that potassium efflux was required for this process. Since NLRP3 inflammasome and AIM2 inflammasome are known to be essential for the activation of caspase-1 by *S. pneumoniae* [18], we examined whether these inflammasomes are involved in pneumococcus-induced pyroptosis of murine microglia. Our results revealed that NLRP3 inflammasome was crucial for caspase-1 activation, subsequent maturation of IL-1β and IL-18 and pyroptotic cell death. However, *S. pneumoniae* failed to activate AIM2 inflammasome (data not shown). In addition, it has been reported that NLRP3 inflammasome contributes to brain injury in pneumococcal meningitis [28]. Indeed, we found that the expression of NLRP3 was significantly increased in the brains of *S. pneumoniae*-infected mice. Caspase-1 activation and subsequent cytokine maturation and production were also induced in the mouse model of pneumococcal meningitis. Collectively, these results suggest that *S. pneumoniae* induces caspase-1-dependent inflammatory cell death and that NLRP3 inflammasome is responsible for the activation of caspase-1 during this process.

During microbial infection, pyroptosis has been known to be a beneficial event by recruiting leukocytes to the site of infection and inhibiting bacterial growth by degradation of invading pathogens [10]. A recent study has suggested that *Staphylococcus aureus* α-hemolysin induces necrotic pulmonary injury in *S. aureus* pneumonia through NLRP3 inflammasome activation and NLRP3-induced necrosis has a role in a protective host response from excessive migration of pulmonary macrophages and pulmonary injury in the pathogenesis of severe *S. aureus* infection [29]. In addition, other study has shown that the basal phenotype of resident macrophages has an effect on the early inflammatory response *via* the inflammasome pathway and subsequent pyroptosis in *S. aureus* infection [30]. By comparing C57BL/6 and DBA/2 mice which are resistant and susceptible to the infection, respectively, pyroptotic process contributes to the secretion of NK-κB-related cytokines and may serve as a protective mechanism by preventing the host from severe outcome of *S. aureus* infection. On the contrary, it has recently been suggested that pyroptosis induced by bacterial infection is detrimental to the host through the mechanisms of microbial immunoevasion. *S. aureus* has been reported to lyse perivascular macrophages which mainly produce neutrophil chemoattractants in inflamed skin [31]. The hemolysin-dependent cell death of perivascular macrophages results in the decreased neutrophil recruitment and provides *S. aureus* a survival advantage. Moreover, excessive pyroptosis has been known to contribute to unfavorable outcomes of various infectious diseases. Previous studies have reported that rapid killing of macrophages prevents appropriate elimination of the invading pathogens and pyroptosis of dendritic cells leads to immunosuppression by impairing cytokine production and antigen presentation [32, 33]. Additionally, pyroptosis causes the excessive production of cytokines and chemokines including TNF-α, IL-1β, IL-6, CCL3, CXCL-1, CXCL-2, etc. in bacterial meningitis which can also result in inflammatory mediator-induced neuronal damage [7]. Since these cytokines and chemokines has been known to mediate immune cell recruitment from peripheral circulation, excessive neutrophils are recruited to the infection sites and extended neutrophilic inflammation causes collateral tissue damage to the brain in bacterial meningitis [34]. In addition, it has been shown that NLRP3 inflammasome plays a central role in brain injury through ATP-dependent lysosomal cathepsin B release in pneumococcal meningitis [28]. Based on these findings, the results of present study suggest that pneumococcus-triggered pyroptosis of microglia is promoted by NLRP3 inflammasome activation and contributes to the pathology of pneumococcal meningitis.

Recent studies have demonstrated that autophagy is an intracellular degradation system that eliminates microbial pathogens by delivering microorganisms to lysosomes [35]. In the current study, we found that autophagy was induced by *S. pneumoniae*. The expression of autophagy-related genes was rapidly increased and LC3 autophagosomes were observed in *S. pneumoniae*-infected BV-2 microglia. Emerging evidence suggests that autophagy is coupled to programmed cell death in response to diverse intracellular microbes, as either a partner or an antagonist [36, 37]. Indeed, several intracellular bacterial pathogens that trigger pyroptosis also induce autophagy [27, 38]. The present data showed that in the presence of autophagy inhibitors, 3-MA, actinomycin D, bafilomycin A1 and siAtg5, the activation of caspase-1 and the subsequent maturation of IL-1β and IL-18 was further induced and that the levels of IL-1β in the culture supernatants were further increased. Additionally, the inhibition of autophagy resulted in an increased level of LDH in the cell culture media. Thus, our data indicate that autophagy transiently protects *S. pneumoniae*-infected microglia from pyroptosis. These results are supported by several studies. One report revealed that caspase-1-dependent cell death occurred more frequently when autophagy was dampened by either 3-MA or an inhibitor of the Atg4 protease in *L. pneumophila*-infected macrophages [27]. Another study also demonstrated that treatment of macrophages with 3-MA enhanced *Shigella*-induced pyroptosis [26]. With regard to the method of regulating pyroptosis by autophagy, several mechanisms have been suggested. One mechanism by which autophagy protects cells from pyroptosis is *via* targeting ubiquitinated inflammasomes for destruction [39]. Other reports have demonstrated that autophagy controls IL-1β secretion by targeting pro-IL-1β for lysosomal degradation and by regulating inflammasome activation [40]. Interestingly, we found that the levels of NF-κB-related cytokines including TNF-α and IL-6 in the culture media were significantly increased in the early stage of pneumococcal infection, whereas cytokine levels were generally maintained during a 4-8 h infection or reduced at 8 h after infection. In contrast, the release of IL-1β was remarkably elevated during pneumococcal infection and high levels of the cytokine was observed at 8 h post-infection. Since autophagy began to increase within 30 min after *S. pneumoniae* infection and remained elevated up to 4 h, autophagy might play a role in degrading pro-IL-1β and regulating low levels of IL-1β in the initial phase of pneumococcal infection. In addition, it is plausible that autophagy contributes to reduce bacterial burden and microbial tissue damage by eliminating invading pathogens. Several reports have suggested that autophagy serves as a cellular defense mechanism in response to various infectious microbes in bacterial meningitis models [41, 42]. Based on these findings, it is plausible that autophagy plays a protective role in the pathology of pneumococcal meningitis.

According to several lines of evidence, increased ROS generation in the mitochondria or other intracellular compartments has been suggested to induce autophagy and pyroptosis [43]. The increased levels of ROS derived from damaged mitochondria enhance autophagy by activating Beclin-1, LC3 II and Atg5-Atg12 protein expression [44]. Pyroptosis can also be triggered by increased oxidative stress to activate infammasome-caspase-1-IL-1β/IL-18 signaling [45]. Recently, it was reported that the activity of NLRP3 inflammasome is negatively regulated by autophagy and positively regulated by ROS [46]. Therefore, it was of interest to investigate the role of ROS in *S. pneumoniae*-induced autophagy, pyroptosis and the cross-talk between them. We found that the production of ROS was inhibited by NAC, a well-known ROS scavenger. In the early phase (4 h) of pneumococcal infection, the expression of autophagy-related genes and the conversion of LC3 I to LC3 II were significantly reduced by treatment with NAC. Additionally, NAC treatment decreased the formation of GFP-LC3 autophagosomes in *S. pneumoniae* D39-infected BV-2 cells. However, contrary to previous studies, the activation of caspase-1 and the maturation of the caspase-1-dependent cytokines, IL-1β and IL-18 were further induced by treatment with NAC. Since autophagy was rapidly induced by *S. pneumoniae* and remained stimulated for 4 h after infection, it is conceivable that autophagy and pyroptosis occur concurrently and that ROS simultaneously activates both pathways at 4 h post-infection. Therefore, we hypothesized that autophagy may be predominantly activated by ROS and may inhibit the activation of caspase-1 and the subsequent maturation of caspase-1-dependent cytokines in the early phase of pyroptosis. We found that inhibited ROS production eventually reduced IL-1β production and the pyroptotic cell death of *S. pneumoniae*-infected microglia. Taken together, the current work demonstrates that the increased levels of ROS by pneumococcal infection stimulate autophagy in the early phase of pyroptosis as a cell survival mechanism and eventually induce inflammatory cell death.

Pneumolysin is a pore-forming cytolysin known as a major virulence factor of *Streptococcus pneumoniae* and is expressed by virtually all clinical isolates of the bacterium. A previous study reported that pneumolysin is an essential factor for the activation of caspase-1 and the consequent secretion of IL-1β and IL-18 in macrophages infected with *S. pneumoniae* [47]. This study showed that the ability to induce the production of IL-1β and IL-18 was severely impaired in macrophages infected with the mutant *S. pneumoniae* strain deficient in pneumolysin (D39ΔPLY) *in vitro*. The study also reported caspase-1 activation in macrophages stimulated with recombinant pneumolysin protein. In addition, other studies have demonstrated that pneumolysin induces the activation of NLRP3 infammasome and caspase-1 [19, 48], and even triggers the release of LDH [18]. Similar to these previous studies, our results revealed that treatment with wild-type pneumolysin resulted in the activation of caspase-1, the maturation of IL-1β and IL-18, and the production of IL-1β in addition to triggering pyroptosis. However, pneumolysin L460D failed to stimulate these processes. The expression of NLRP3 was also increased by pneumolysin treatment indicating the involvement of NLRP3 infammasome in pneumolysin-induced pyroptosis of murine microglia. In addition, caspase-1 activation and subsequent cytokine maturation were significantly inhibited by D39ΔPLY infection. Furthermore, the levels of IL-1β and LDH in the culture media of D39ΔPLY-infected cells were severely reduced. In a mouse model of pneumococcal meningitis, we found that the activation of capsase-1 and the maturation of subsequent cytokines were less induced in the brains of D39ΔPLY-challenged mice than in D39-challenged mice. Moreover, significant reduced levels of IL-1β were observed in the brain homogenates of D39ΔPLY-infected mice. In agreement with our data, it has previously been demonstrated that the lack of pneumolysin is associated with a better clinical course and less brain inflammation in murine pneumococcal meningitis [28]. Based on these findings, our results suggest that pneumolysin plays an important role in *S. pneumoniae*-induced pyroptosis of murine microglial cells and in murine pneumococcal meningitis.

In conclusion, the results of the present study suggest that *S. pneumoniae* induces pyroptosis through NLRP3 inflammasome activation in murine microglia and in murine pneumococcal meningitis. We also demonstrate that autophagy is stimulated and protects pneumococcus-infected microglia from pyroptotic cell death. These findings provide further evidence for a link between autophagy and pyroptosis in pneumococcal infection.

## MATERIALS AND METHODS

### Cell lines and reagents

The mouse microglial cell line, BV-2, was kindly provided by Prof. Choon-gon Jang at Sungkyunkwan University [49]. The rat glioma cell line (C6) was obtained from American Type Culture Collection (Manassas, Virginia, USA). The cells were propagated in Dulbecco's Modified Eagle's Medium supplemented with 10% heat-inactivated fetal bovine serum and antibiotics (100 IU/ml of penicillin and 100 μg/ml of streptomycin). Cultures were maintained in a humidified incubator at 37°C with 5% CO_2_. FBS, DMEM, penicillin G, and streptomycin were obtained from Life Technologies Inc. (Carlsbad, CA, USA). Dihydroethidium 2′7′-dichlorodihydrofluorescein diacetate (H_2_DCFDA) was obtained from Molecular Probes (Eugene, OR, USA). Caspase-1, Caspas-1 p10, IL-18, Beclin-1 and β-actin antibodies were obtained from Santa Cruz Biotechnology (Dallas, TX, USA). NLRP3 antibody was obtained from Adipogen (San Diego, CA, USA). IL-1β antibody was obtained from R&D Systems (Minneapolis, MN, USA). Atg5, LC3 and Iba-1 antibodies were obtained from Novus Biologicals (Littleton, CO, USA). Unless otherwise indicated, all the chemicals used in this study were purchased from Sigma Chemical Co. (St. Louis, MO, USA).

### Bacterial strains

*Streptococcus pneumoniae* D39 (serotype 2), its isogenic pneumolysin-deficient mutant (D39ΔPLY), and TIGR4 (serotype 4) were provided by Prof. David E. Briles at the University of Alabama at Birmingham [50] and were cultured in Todd-Hewitt media containg (per liter) 30 g of Todd Hewitt Broth, 0.5% Yeast extract, and 1.5% Agar. The broth was sterilized for 15 min at 121°C. The pneumococcal growth was cultured as described previously [50, 51]. The pneumococcus was cultured at 37°C overnight on THY blood agar (supplemented with erythromycin where appropriate). Subsequently, the pneumococcus grown on THY agar plate was transferred into THY broth and cultures were grown in broth at 37°C without aeration. Cultures to be frozen were first brought to 10 to 15% glycerol and then stored at −70°C.

### Mice

Male C57BL/6 at 6-8 weeks of age were purchased from DBL (Korea) and acclimatized under controlled conditions for 2 weeks before the experiment. The animals were housed in cages located in temperature controlled rooms with a 12:12 h light-dark cycle, and received water and food *ad libitum*. Each experimental group consisted of ten mice and the animal experiment was conducted once. All animal procedures were approved by the Sungkyunkwan University Animal Care Committee.

### Mouse meningitis model

A well-characterized mouse model of pneumococcal meningitis was used with a little modification [52]. Briefly, meningitis was induced by intracerebral ventricular (icv) injection of 10 μl bacterial suspension containing 10^6^ CFU/ml *S. pneumoniae* serotype 2 strain D39, its isogenic pneumolysin mutant D39ΔPLY or serotype 4 strain TIGR4 into a soft spot in the cranium located 1∼2 mm in front of the coronal suture, and 1 mm on either side of the sagittal suture, which runs vertically down the midline of the cranium. For rescue experiments, a combination of D39ΔPLY (10^6^ CFU/ml) and PLY (200 ng PLY / g mouse) was injected into mouse brains [53]. After 24 h of pneumococcal infection, animals were sacrificed by CO_2_ inhalation.

### Purification of recombinant pneumolysin

The pneumolysin (PLY) gene was amplified by PCR with primers of PLY (Table 1) that incorporated BamHI and XhoI restriction enzyme sites. The PCR products were digested with BamHI and XhoI enzymes and cloned to the plasmid pET32b(+). After transformation, the E. coli BL21 pET32b-PLY was grown in LB media supplemented with kanamycin (20 μg/ml) at 37°C in a shaking incubator until an OD_600_ of 0.6-0.8 was reached. The protein was overexpressed by providing IPTG (final concentration of 0.5 mM) to the culture. Then the bacteria were incubated at 25°C in a shaking incubator overnight. Cells were harvested by centrifugation at 4,000 rpm for 15 min at 4° C. The pellet was resuspended in 10 ml of lysis buffer (50 mM Tris-HCl pH 7.5, 10% glycerol, 1 mM DTT, 1 mM PMSF). The suspension was frozen and thawed 3 times, sonicated, and centrifuged at 13,000 rpm for 20 min at 4° C. The supernatant was used for histidine tagged PLY protein purification using a Ni-NTA column. The detoxified derivative of pneumolysin, pneumolysin L460D was provided by Prof. James C. Paton at the University of Adelaide [54].

**Table 1:** Sequence of primers used in this study

Primer	Sequence (5′ → 3′)
PLY Forward	GGGCCCGGATCCGATGGCAAATAAAGCAGTAAATGAC
PLY Reverse	GGCCCGCTCGAGCTAGTCATTTTCTACCTTATCCTC

### Immunoblot analysis

The lysates of *S. pneumoniae*-infected or pneumolysin-treated cells and the homogenates of the brain were prepared using RIPA buffer (150 mM NaCl, 50 mM Tris-HCl pH 8.0, 5 mM EDTA, 1% sodium deoxycholate, 0.1% SDS and 1% Triton X-100) supplemented with protease inhibitors before use. Supernatants of *S. pneumoniae*-infected cells were also collected, concentrated and precipitated by trichloroacetate. Protein concentrations were quantified using the DC protein Assay. Aliquots (15∼30 μg of protein) of cell lysates and brain homogenates were resolved by SDS-PAGE, followed by the transfer to NC/PVDF membrane. After blocking with 5% skim milk in Tris-buffered saline containing 0.05% Tween-20 (TBST) for 2 h at room temperature, the membrane was probed overnight at 4°C with primary antibody diluted in phosphate-buffered saline containing 0.1% Tween-20 (PBST). Following several washes with TBST, the membrane was incubated at room temperature for 1 h with the appropriate horseradish peroxidase-conjugated secondary antibody diluted in PBST. After the final washes, the immunocomplexes were developed using Western blotting detection reagent. After measuring the intensity of each band by densitometry using the image processing software Image J, the relative intensities were calculated by normalizing to β-actin from the corresponding sample.

### ELISA

The levels of secreted IL-1β in the culture supernatants of *S. pneumoniae*-infected BV-2 cells and in mouse brain homogenates were determined by two-site sandwich ELISA according to the manufacturer's instructions. The ELISA Kit for IL-1β was purchased from Biolegend Inc. (San Diego, CA, USA).

### Detection of LDH release

The levels of lactate dehydrogenase (LDH) in culture supernatants were measured using the CytoTox96 LDH-release kit from Promega Co. (Fitchburg, WI, USA) according to the manufacturer's instructions. The percentage of LDH release was calculated using the following formula.

$$\text{Percentage of LDH release}=100\times \frac{\left(\text{experimental LDH release}-\text{spontaneous LDH release}\right)}{\left(\text{maximal LDH release}-\text{spontaneous LDH release}\right)}$$

### RNA interference

Nonsilencing control siRNA and the siRNAs targeting mouse NLRP3 and Atg5 were chemically synthesized (Genolution Parmaceuticals Inc., Seoul, Korea) (Table 2). To knockdown endogenous NLRP3 and Atg5, cells were transiently transfected with siRNAs for 24 h at a final concentration of 100 nM and 50nM, respectively, using the iN-fect™ *in vitro* Transfection Reagent (iNtRON Biotechnology, Inc. Sungnam, Korea) according to the manufacturer's protocol.

**Table 2:** Sequences of siRNAs

siRNA		Sequence (5′ → 3′)
siNLRP3	Sense	GGUGAAAUGUACUUAAAUCUU
siNLRP3	Antisense	GAUUUAAGUACAUUUCACCUU
siAtg5	Sense	AACUUGCUUUACUCUCUCUAUCAUU
siAtg5	Antisense	UGAUAGAGAGAGUAAAGCAAGUUUU
Control	Sense	GUGCACAUGAGUGAGAUUUUU
Control	Antisense	AAAUCUCACUCAUGUGCACUU

### Detection of autophagy by GFP-LC3

BV-2 cells were transfected with GFP-LC3 for 24 h and infected with *S. pneumoniae* at an MOI of 100 for 4 h to induce autophagy. Cells with GFP-LC3^+^ dots were visualized and counted with a LSM 700 confocal microscope (ZEISS, Seoul, Korea). Cells were considered positive if they had more than three large GFP-LC3^+^ dots. Data are presented as a percentage of the total number of GFP^+^ cells visualized.

### Measurement of ROS production

Intracellular reactive oxygen species (ROS) generation by pneumococcal infection was assessed using the cell permeable, non-polar H_2_O_2_ sensitive dye 5-(and-6)-chlromethyl-2′, 7′- dichlorodihydrofluorescein diacetate (CM-H_2_DCFDA). Briefly, BV-2 cells were incubated for 2 h with 10 mM of N-Acetyl Cysteine (NAC) followed by *S. pneumoniae* infection for 2 h. The cells were subsequently treated with CM-H_2_DCFDA (50 μM) for 5 min at 37°C, washed twice with PBS, and the fluorescent intensity of the cells was measured using FACS Canto II (BD Biosciences, San Jose, CA, USA).

### Immunohistochemistry and immunofluorescence

For immunofluorescence staining, brains were harvested and placed in a cryo-mold on a thin layer of Optimal Cutting Temperature (OCT) embedding media (Sakura, Japan), and then covered completely with OCT. OCT-embedded samples were snap-frozen by floating on liquid nitrogen. Frozen blocks were further stored at −80°C until sectioned. Frozen tissues were cut into 10 μm sections, mounted on poly-lysine coated slides, and allowed to air-dry overnight at room temperature prior to staining. The sections were washed three times with PBS, incubated with blocking buffer (10 mM PBS with 10% normal goat serum and 0.3% Triton X-100) for 1 h at room temperature, and then incubated overnight at 4°C with primary antibodies against caspase-1 (1:50) and Iba-1 (1:50) in PBS containing 1% normal goat serum and 0.3% Triton X-100. Anti-rabbit FITC (Santa Cruz Biotechnology, Dallas, TX, USA) was used for caspase-1 and caspase-1 p10 and anti-goat Texas Red (Santa Cruz Biotechnology, Dallas, TX, USA) was used for Iba-1. Nuclei were stained with DAPI. Processed sections were analyzed using a LSM700 confocal microscope (ZEISS, Seoul, Korea).

### Statistical analysis

All experiments were performed at least three times (unless otherwise indicated), and the results of one representative experiment are shown. For comparisons between two groups, the Student's *t* test was used. Multi-group comparisons of mean values were analyzed by one-way ANOVA. Experimental differences were considered statistically significant when a P value was less than 0.05.

## SUPPLEMENTARY MATERIAL FIGURES


