# Supplementary material for: Streptococcus pneumoniae induces pyroptosis through the regulation of autophagy in murine microglia
Source: Oncotarget. 2015 Dec 13;6(42):44161–78. doi: 10.18632/oncotarget.6592 (PMC4792549; doi:10.18632/oncotarget.6592)
Supplement: Supplementary file 1 [file oncotarget-06-44161-s001.pdf]

Streptococcus pneumoniae induces pyroptosis through the regulation of autophagy in murine microglia

Supplementary Material

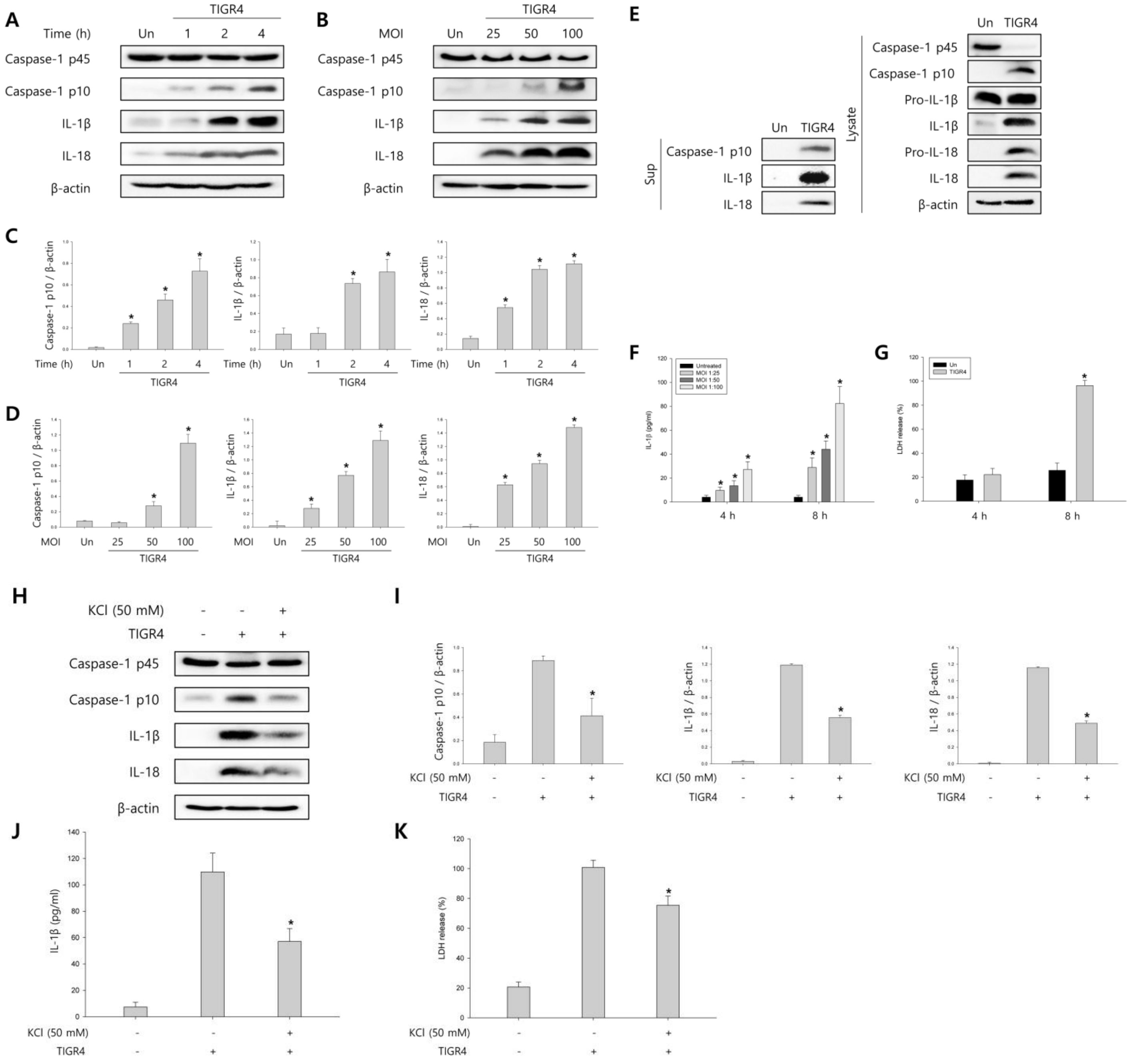

Supplementary Figure S1: *S. pneumoniae* TIGR4 induces caspase-1 activation and pyroptosis in BV-2 microglial cells. (**A-D**) BV-2 microglial cells were left uninfected or were infected with *S. pneumoniae* TIGR4 at various MOI of 25, 50 and 100 for 1, 2 and 4 h. Cell lysates were subjected to Western blot analysis to detect caspase-1, IL-1β and IL-18. The levels of each protein expression are in arbitrary units, and data are normalized to the respective amount of β-actin protein. These results are representative of three independent experiments with similar results. *$p < 0.05$, significantly different from uninfected control. (**E**) BV-2 cells were left uninfected or were infected with TIGR4 at an MOI of 100 for 4 h. Culture

supernatants were concentrated by trichloroacetate precipitation. Concentrated supernatants and cell lysates were subjected to Western blot analysis using antibodies specific for caspase-1, IL-1β, IL-18 and β-actin. β-actin was used as a loading control. These results are representative of three independent experiments. (**F**) Microglial cells were infected with TIGR4 at various MOI of 25, 50 and 100 for 4 h and 8 h. The levels of IL-1β in the culture supernatants were determined by ELISA. Data are expressed as the mean ± S.E.M. of 3 experiments. *$p < 0.05$, significantly different from uninfected control. (**G**) BV-2 microglial cells were infected with TIGR4 at an MOI of 100 for 4 h and 8 h. LDH release into the culture media is shown as a percentage of LDH release. Data are expressed as the mean ± S.E.M. of 3 experiments. *Significantly different from uninfected cells ($P<0.05$). (**H-K**) Potassium efflux is required for *S. pneumoniae*-induced pyroptosis. (**H** and **I**) BV-2 microglial cells were infected with *S. pneumoniae* TIGR4 at an MOI of 100 in the presence or absence of 50 mM of KCl for 4 h. Cell lysates were subjected to Western blot analysis to detect caspase-1 and caspase-1-dependent cytokines. The levels of each protein expression are in arbitrary units, and data are normalized to the respective amount of β-actin protein. These results are representative of three independent experiments with similar results. *$p < 0.05$, significantly different from *S. pneumoniae*-infected control. (**J** and **K**) BV-2 cells were infected with TIGR4 at an MOI of 100 in the presence or absence of 50 mM of KCl for 8 h. The levels of IL-1β in the culture supernatants were determined by ELISA (**J**). LDH release into the culture media is shown as a percentage of LDH release (**K**). Data are expressed as the mean ± S.E.M. of 3 experiments. *$p < 0.05$, significantly different from *S. pneumoniae*-infected control.

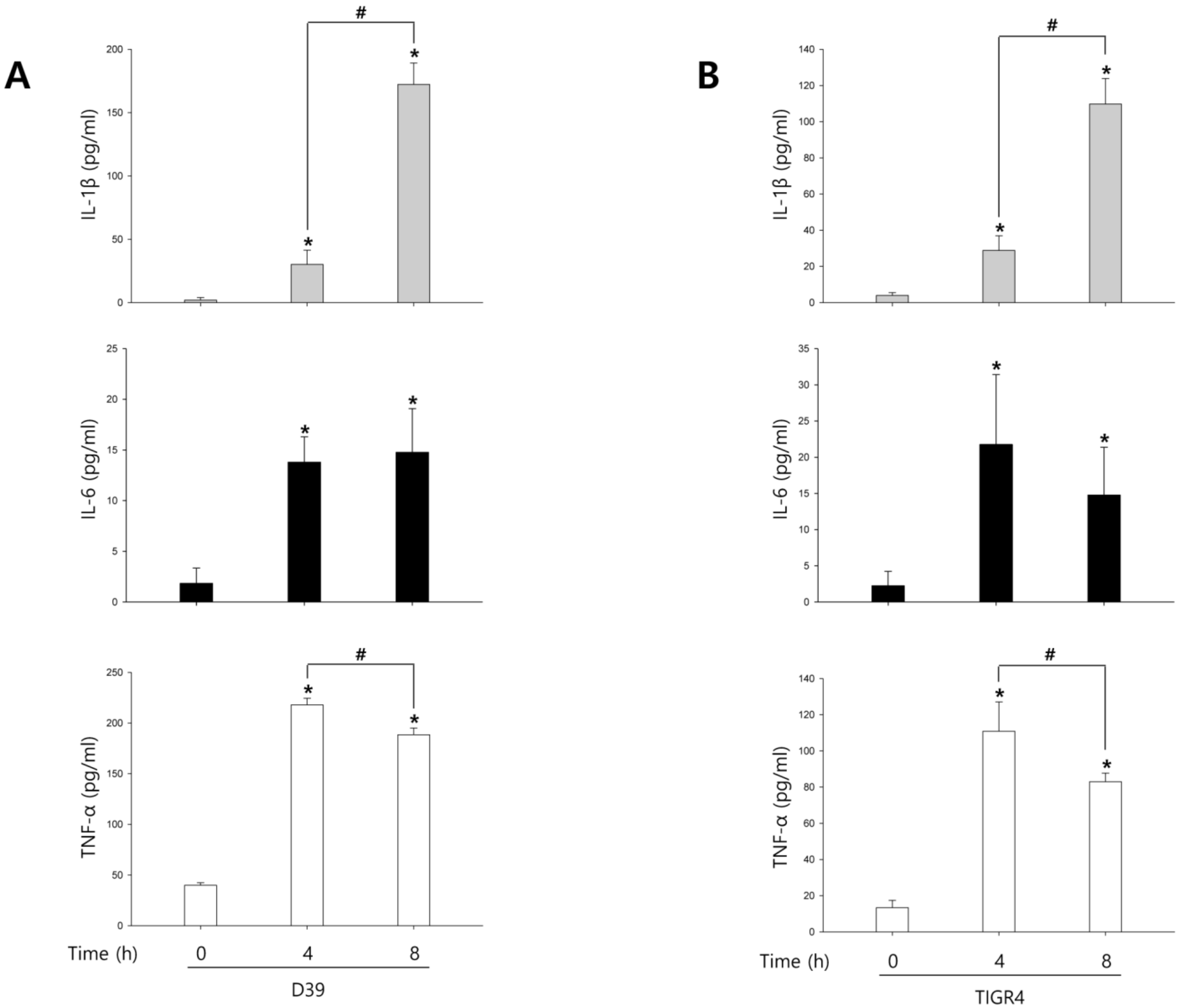

Supplementary Figure S2: ***S. pneumoniae* induces the production of IL-1β, IL-6 and TNF-α.** BV-2 microglial cells were infected with D39 (**A**) or TIGR4 (**B**) at an MOI of 100 for 4 h and 8 h. The levels of IL-1β, IL-6 and TNF-α in the culture supernatants were determined by ELISA. Data are expressed as the mean ± S.E.M. of 3 experiments. *$p < 0.05$, significantly different from uninfected control. $^{\#}p < 0.05$, Statistical difference with respect to time of pneumococcal infection.

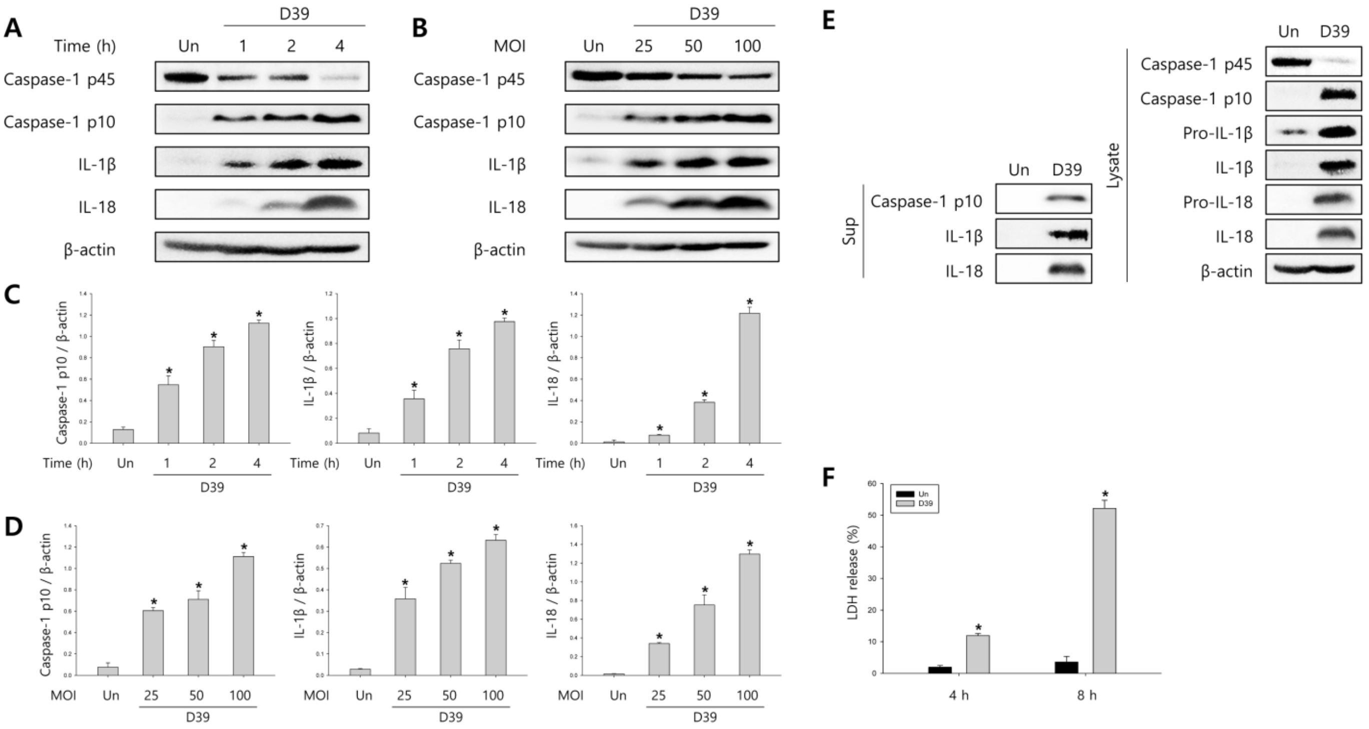

Supplementary Figure S3: ***S. pneumoniae*** **D39 induces caspase-1 activation and pyroptosis in C6 glioma cells.** (**A-D**) C6 glioma cells were left uninfected or were infected with *S. pneumoniae* D39 at various MOI of 25, 50 and 100 for 1, 2 and 4 h. Cell lysates were subjected to Western blot analysis to detect caspase-1, IL-1β and IL-18. The levels of each protein expression are in arbitrary units, and data are normalized to the respective amount of β-actin protein. These results are representative of three independent experiments with similar results. *p < 0.05, significantly different from uninfected control. (**E**) C6 cells were left uninfected or were infected with D39 at an MOI of 100 for 4 h. Culture supernatants were concentrated by trichloroacetate precipitation. Concentrated supernatants and cell lysates were subjected to Western blot analysis using antibodies specific for caspase-1, IL-1β, IL-18 and β-actin. β-actin was used as a loading control. These results are representative of three independent experiments. (**F**) C6 glioma cells were infected with D39 at an MOI of 100 for 4 h and 8 h. LDH release into the culture media is shown as a percentage of LDH release. Data are expressed as the mean ± S.E.M. of 3 experiments. *Significantly different from uninfected cells (P<0.05).

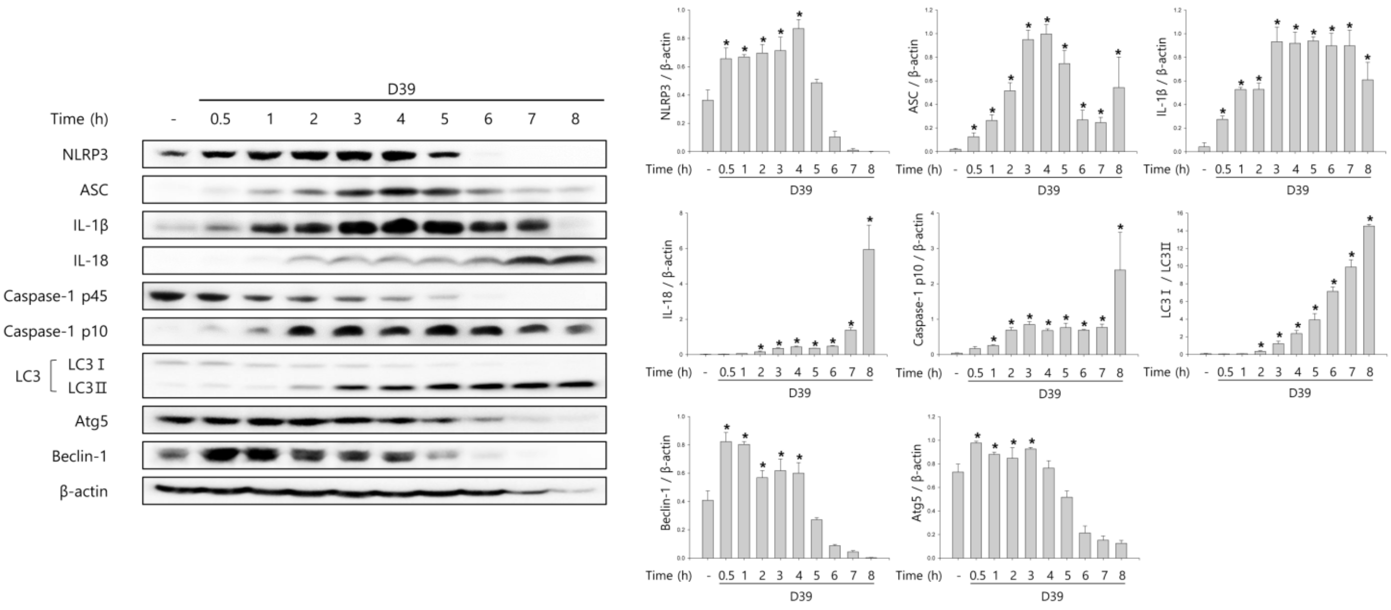

Supplementary Figure S4: Time-dependent expression of inflammasome-related proteins, caspase-1, caspase-1-dependent cytokines and autophagy-related proteins. BV-2 microglial cells were infected with D39 at an MOI of 100 for indicated times. Cell lysates were subjected to Western blot analysis. β-actin was used as a loading control. These results are representative of three independent experiments with similar results. *$p < 0.05$, significantly different from uninfected control.

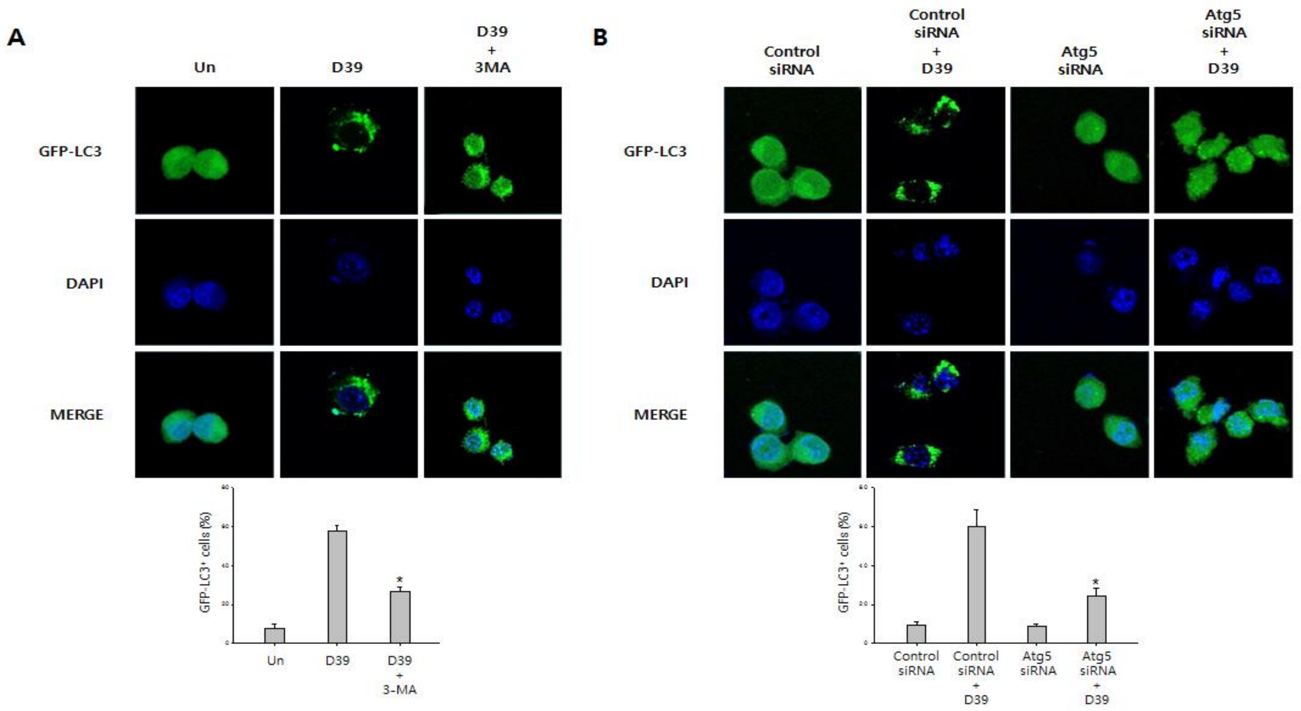

Supplementary Figure S5: Autophagy is induced by *S. pneumoniae*. (**A**) BV-2 cells were transfected with GFP-LC3 for 24 h and infected with *S. pneumoniae* D39 in the presence or absence of 3-MA (1 mM) at an MOI of 100 for 4 h. (**B**) BV-2 cells were co-transfected with GFP-LC3 and siRNA (control siRNA or Atg5 siRNA) for 24 h and infected with *S. pneumoniae* D39 at an MOI of 100 for 4 h. GFP-LC3 expressing cells were visualized with a fluorescence microscope and the results are presented as quantification of GFP-LC3^{+} dots. These results are representative of three independent experiments with similar results. *$p < 0.05$, significantly different from *S. pneumoniae* D39-infected control.

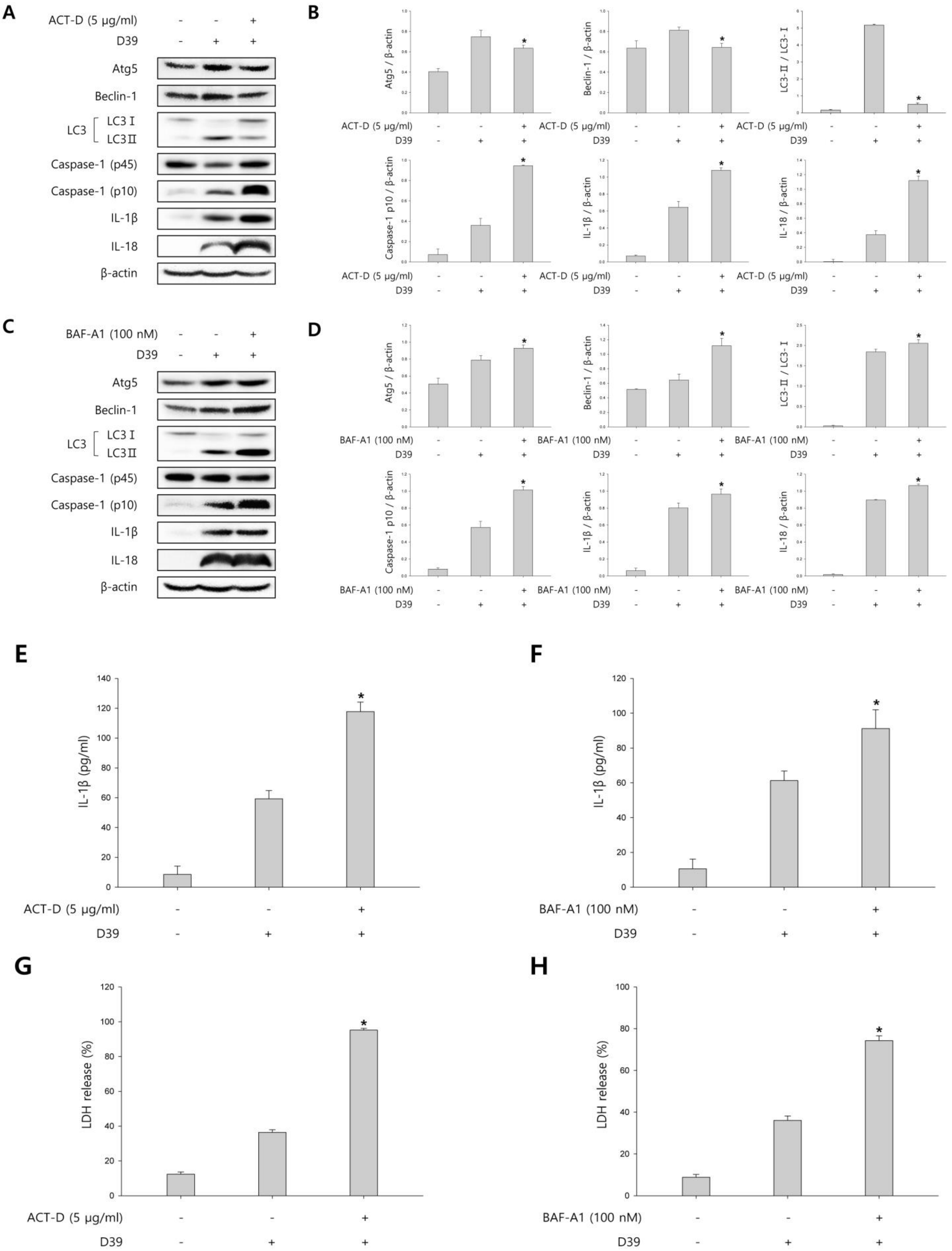

A
ACT-D (5 μg/ml)
D39
Atg5
Beclin-1
LC3
LC3 I
LC3 II
Caspase-1 (p45)
Caspase-1 (p10)
IL-1β
IL-18
β-actin
B
Atg5 / β-actin
Beclin-1 / β-actin
LC3-II / LC3-I
Caspase-1 p10 / β-actin
IL-1β / β-actin
IL-18 / β-actin
C
BAF-A1 (100 nM)
D39
D
E
IL-1β (pg/ml)
F
G
LDH release (%)
H

Supplementary Figure S6: Autophagy inhibits *S. pneumoniae*-induced caspase-1 activation and pyroptosis. (**A** and **B**) BV-2 cells were left uninfected or were infected with D39 at an MOI of 100 for 2 h (Atg5 and Beclin-1) and 4 h in the presence or absence of actinomycin D (ACT-D, 5 µg/ml). Cell lysates were subjected to Western blot analysis. The levels of each protein expression are in arbitrary units, and data are normalized to the respective amount of β-actin protein. The LC3 I and LC3 II bands were quantified by densitometry. These results are representative of three independent experiments with similar results. *$p < 0.05$, significantly different from *S. pneumoniae* D39-infected control. (**C** and **D**) BV-2 cells were left uninfected or were infected with D39 at an MOI of 100 for 2 h (Atg5 and Beclin-1) and 4 h in the presence or absence of bafilomycin A1 (BAF-A1, 100 nM). Cell lysates were subjected to Western blot analysis. The levels of each protein expression are in arbitrary units, and data are normalized to the respective amount of β-actin protein. The LC3 I and LC3 II bands were quantified by densitometry. These results are representative of three independent experiments with similar results. *$p < 0.05$, significantly different from *S. pneumoniae* D39-infected control. (**E** and **G**) BV-2 microglial cells were left uninfected or were infected with D39 at an MOI of 100 for 8 h in the presence or absence of actinomycin D (ACT-D, 5 µg/ml). (**F** and **H**) BV-2 cells were left uninfected or were infected with D39 at an MOI of 100 for 8 h in the presence or absence of bafilomycin A1 (BAF-A1, 100 nM). The levels of IL-1β in the culture supernatants were determined by ELISA (**E** and **F**). The levels of LDH in the culture media are shown as a percentage of LDH release (**G** and **H**). These results are representative of three independent experiments with similar results. *$p < 0.05$, significantly different from *S. pneumoniae* D39-infected control.

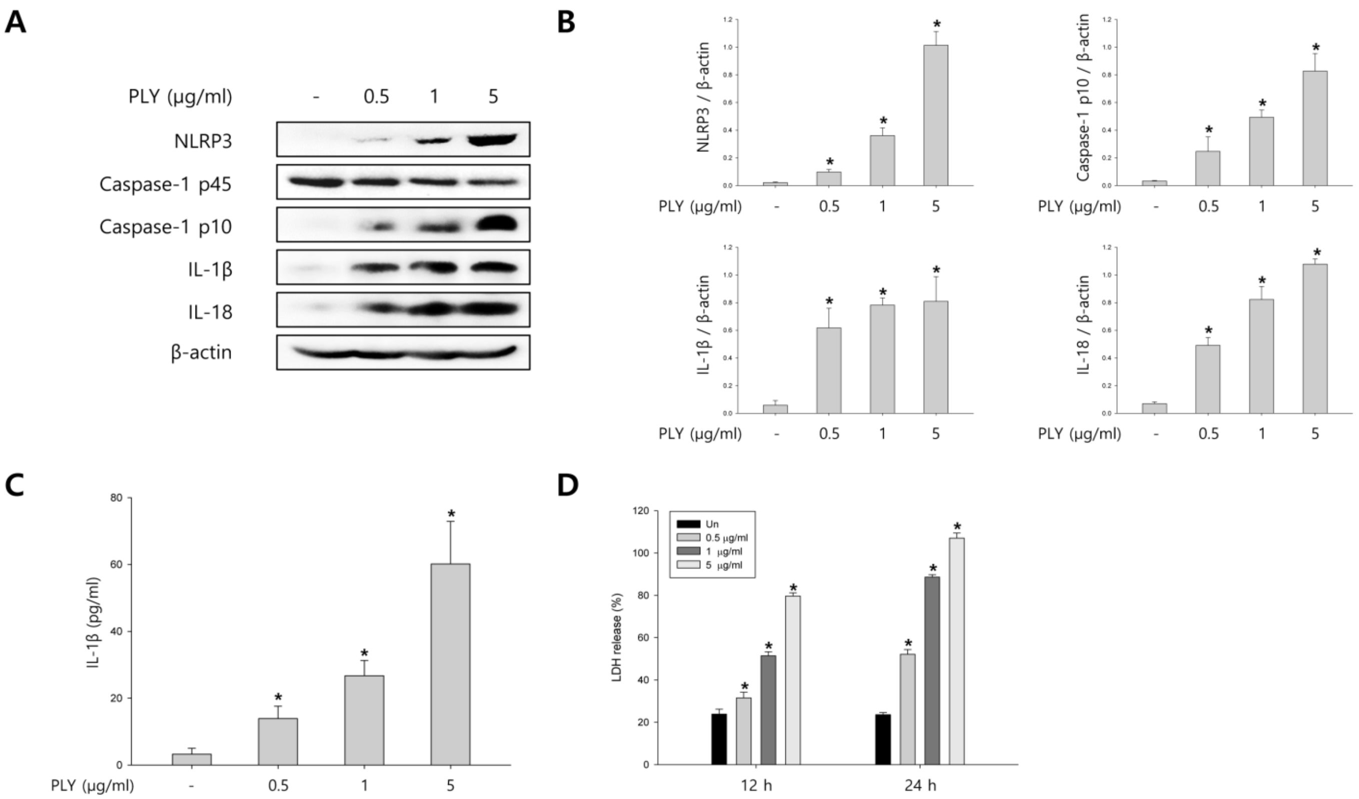

Supplementary Figure S7: Pneumolysin induces caspase-1 activation and triggers pyroptosis. (**A** and **B**) BV-2 microglial cells were treated with 0.5, 1 and 5 μg/ml of pneumolysin (PLY) for 6 h (NLRP3) and 12 h. Cell lysates were subjected to immunoblot analysis. The levels of each protein expression are in arbitrary units, and data are normalized to the respective amount of β-actin protein. These results are representative of three independent experiments with similar results. *$p < 0.05$, significantly different from untreated control. (**C**) BV-2 cells were treated with 0.5, 1 and 5 μg/ml of PLY for 24 h. The levels of IL-1β in the culture supernatants were determined by ELISA. These results are representative of three independent experiments with similar results. *$p < 0.05$, significantly different from untreated control. (**D**) Microglial cells were treated with 0.5, 1 and 5 μg/ml of PLY for 12 h and 24 h. The levels of LDH in the culture supernatant are shown as a percentage of LDH release. These results are representative of three independent experiments with similar results. *$p < 0.05$, significantly different from untreated control.

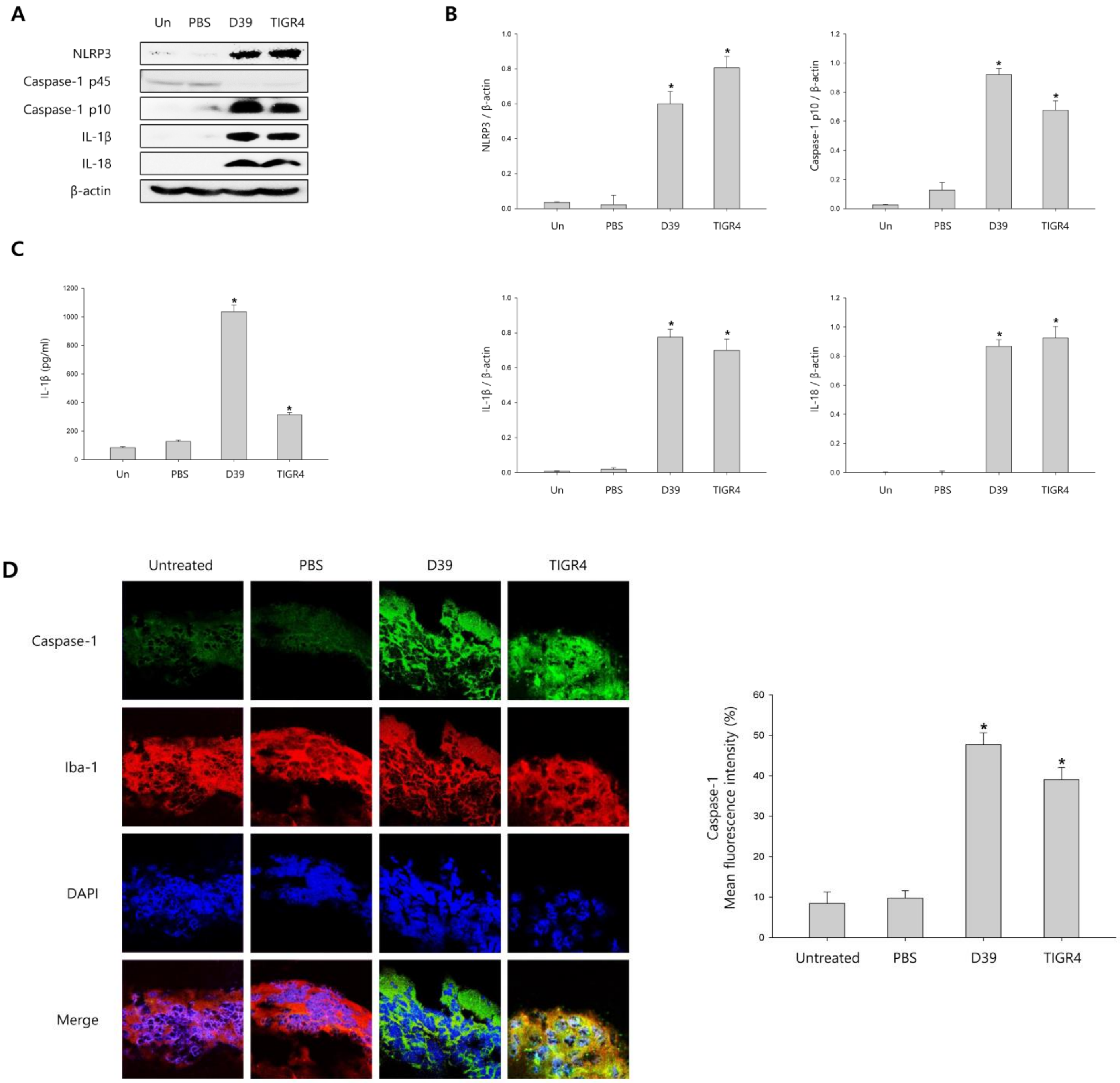

Supplementary Figure S8: *S. pneumoniae* induces caspase-1 activation in murine pneumococcal meningitis. (**A** and **B**) C57BL/6 mice were challenged with control PBS, *S. pneumoniae* D39 or *S. pneumoniae* TIGR4 via intracerebral ventricular injection. The protein expression of NLRP3, Caspase-1, IL-1β and IL-18 in brain homogenates was determined by Western blot analysis. The levels of each protein expression are in arbitrary units, and data are normalized to the respective amount of β-actin protein. These results are representative of three independent experiments with similar results. *$p < 0.05$, significantly different from PBS-treated control. (**C**) The levels of IL-1β in brain homogenates were determined by ELISA. These results are representative of three independent experiments with similar results. *$p < 0.05$, significantly different from PBS-treated control. (D) Immunohistochemical analysis of caspase-1 in *S. pneumoniae*-infected mouse brains. Sections were stained for caspase-1 (FITC) and the glial cell marker, Iba-1 (Texas Red) (x400).
